# Phylodynamics and Coat Protein Analysis of Babaco Mosaic Virus in Ecuador

**DOI:** 10.3390/plants11131646

**Published:** 2022-06-22

**Authors:** Francisco Mosquera-Yuqui, Francisco J. Flores, Eduardo A. Moncayo, Brighitte A. Garzón-Proaño, Miguel A. Méndez, Fiama E. Guevara, Diego F. Quito-Avila, William Viera, Juan F. Cornejo-Franco, Andrés R. Izquierdo, Carlos Noceda

**Affiliations:** 1 Grupo de Investigación y Desarrollo de la Biotecnología BioSin-Biociencias, Quito 170801, Ecuador; eamoncayo@utpl.edu.ec; 2 Unidad de Citometría de Flujo y Biología Molecular, Departamento de Apoyo Diagnóstico, Hospital Oncológico Solón Espinosa Ayala, SOLCA Núcleo de Quito, Quito 170138, Ecuador; 3 Departamento de Ciencias de la Vida y la Agricultura, Universidad de las Fuerzas Armadas-ESPE, Sangolquí 171103, Ecuador; feguevara@espe.edu.ec (F.E.G.); arizquierdo@espe.edu.ec (A.R.I.); cmnoceda@espe.edu.ec (C.N.); 4 Centro de Investigación en Alimentos CIAL, Universidad UTE, Quito 170147, Ecuador; 5 Departamento de Química, Universidad Técnica Particular de Loja (UTPL), Loja 110107, Ecuador; 6 Instituto de Simulación Computacional, Universidad San Francisco de Quito, Cumbayá 170901, Ecuador; bagarzonp94@gmail.com (B.A.G.-P.); mmendez@usfq.edu.ec (M.A.M.); 7 Centro de Investigaciones Biotecnológicas del Ecuador (CIBE), Escuela Superior Politécnica del Litoral (ESPOL), Guayaquil 090112, Ecuador; dquito@espol.edu.ec (D.F.Q.-A.); jcornejo@espol.edu.ec (J.F.C.-F.); 8 Fruit Program, National Institute of Agricultural Research (INIAP), Quito 170184, Ecuador; william.viera@iniap.gob.ec; 9 Centro de Nanociencia y Nanotecnología CENCINAT, Universidad de las Fuerzas Armadas-ESPE, Sangolquí 171103, Ecuador; 10 Facultad de Ingeniería, Universidad Estatal de Milagro (UNEMI), Guayas 091050, Ecuador; 11 Non-Institutional Competence Focus (NICFocus) ‘Functional Cell Reprogramming and Organism Plasticity’ (FunCROP), Coordinated from Foros de Vale de Figueira, 7050-704 Montemor-o-Novo, Portugal

**Keywords:** babaco, babaco mosaic virus, phylodynamics, coat protein

## Abstract

Babaco is a fast-growing herbaceous shrub with great commercial potential because of the organoleptic properties of its fruit. Babaco mosaic virus (BabMV) is a potexvirus in the family *Alphaflexiviridae* affecting babaco in all the provinces that produce this crop in Ecuador. BabMV was recently described but it has been affecting babaco for decades and, since many potexviruses are serologically indistinguishable, it may have been previously misidentified as papaya mosaic virus. Based on the coat protein (CP) gene, we aimed to study the distribution and epidemiological patterns of BabMV in babaco and chamburo over the years and to model its three-dimensional structure. Sequences of the CP were obtained from thirty-six isolates from plants collected in the main babaco-producing provinces of Ecuador between 2016 and 2021. The evolution rate of BabMV was estimated at 1.21 × 10^−3^ nucleotide substitutions site^−1^ year^−1^ and a time of origin of the most recent common ancestor around 1958.80. From molecular dynamics simulations, compared to other proteins of BabMV—RDRP, TGB1, and Alkb domain—the CP exhibited a higher flexibility with the C and N terminals as the most flexible regions. The reconstructed viral distribution provides dispersion patterns which have implications for control approaches of BabMV.

## 1. Introduction

Babaco (*Vasconcellea* × *heilbornii*) is an exotic fruit native to the mountainous region of Ecuador, originating from a natural cross between toronche (*Vasconcellea stipulata*) and chamburo (*Vasconcellea pubescens*). Currently, it is cultivated as far north as Guernsey and as far south as New Zealand [1] where new hybrids, obtained by selective breeding between *Vasconcellea* species, have gained attention in the last decades. Papaya (*Carica papaya*) is the most commercially important relative to these *Vasconcellea* species [2].

Large-scale production of babaco is currently carried out in Ecuador and New Zealand to satisfy domestic consumption and external demand [3]. This fruit crop is grown in the provinces of Imbabura, Pichincha, Tungurahua, Azuay, and Loja in Ecuador [4]; commercial babaco plants produce 60–80 fruits per tree every year. While 2-year-old fields in Ecuador yield between 40 and 60 ton ha^−1^ year^−1^, yields of up to 100 ton ha^−1^ year^−1^ have been reported from New Zealand [5,6]. The fruits are rich in vitamin C, low in sugar, and have a fragrant, flavorful and juicy pulp covered by an edible skin [7]. Processed babaco and chamburo products include marmalade, juice concentrate, jam, syrup, and dehydrated fruit. Babaco is also a source of proteolytic enzymes as the proteolytic activity of the latex from green fruit was reported as equivalent or higher than the one from commercial papain [8]. Taken together, babaco has great potential to become a major product in the fruit market. However, the crop is seriously affected by pests and diseases caused by fungi, bacteria, nematodes, and several viruses including babaco mosaic virus (BabMV) [9,10].

Based on demarcation criteria and its complete genome sequence, BabMV was proposed as a new member of the genus *Potexvirus* [9]. This pathogen may have been previously reported from Italy in 1989 as babaco yellow mosaic virus [9,11], which was later observed using electron microscopy [12]. No further studies have been made on the structural characteristics of the virus. Aminoacid sequence identity between BabMV and its closest relatives, papaya mosaic virus (PapMV) and alternanthera mosaic virus (AltMV), is 70% for the polymerase and 72% for the coat protein (CP) [9]; however, these viruses were serologically indistinguishable [9,11]. There are no structures available for the CP of viruses related, but bioinformatic modeling can help to understand how these divergent sequences, belonging to different potexvirus species, can conserve a structure that is indistinguishable by antibodies. To date, babaco plants showing leaf mottling and yellow mosaic have been found in most of the provinces of Ecuador where this crop is cultivated.

The main premise in viral epidemic processes is that they leave a quantifiable imprint on genomes over time. In rapidly mutating viruses such as RNA viruses, the evolution of their genome occurs at the same time with its geographic dispersal; this phylodynamic process can be modeled with genomic data using phylogeographic analyzes [13]. Understanding how BabMV has spread throughout the country will help to determine strategies to control its dissemination and will provide valuable information for the establishment of a virus-free babaco plant production program. The purpose of this study was to reconstruct the phylodynamics of BabMV in Ecuador and to analyze bioinformatically its CP. 

## 2. Results

### 2.1. Nucleotide Sequences and Recombination Analyses 

Multiple sequence alignment of 36 BabMV CP sequences, where the complete open reading frame consists of 654 bp corresponding to 217 aa, was carried out. Pairwise analysis revealed the highest nucleotide variation between an isolate from chamburo sampled in Azuay and the other from babaco collected in Pichincha (87.25%, MT240513-OL771200). At amino acid level, the highest variability was observed between two isolates from Tungurahua, sampled the same date, (91.84%, MT240497-MT240499). Based on the ICTV guidelines for species demarcation in the genus *Potexvirus* (isolates of different species have less than 72% nt identity or 80% aa identity) [14], all the isolates belonged to BabMV. Because recombination can affect the topology of a phylogenetic tree, potential recombination events were assessed; however, no recombinant sequences were detected.

### 2.2. Phylogenetic Analysis and Geographical Spread of BabMV

**Figure 1:**
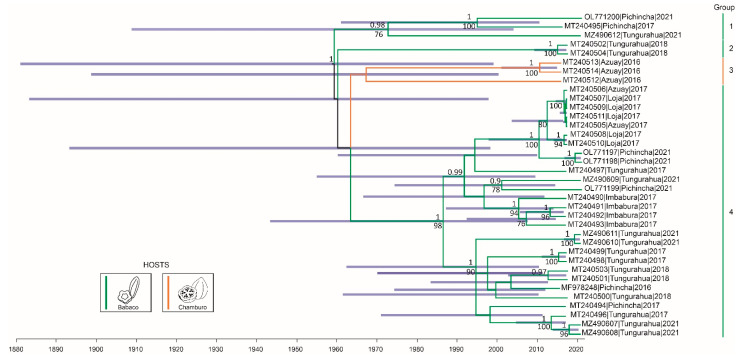
Bayesian phylogenetic tree under strict molecular clock and Bayesian skyline model inferred from 36 nucleotide sequences of the coat protein of babaco mosaic virus. Branches are color-coded according to the host species. Posterior probabilities are shown above the nodes, and bootstrap support values from maximum-likelihood inference are shown below the nodes. Only posterior probability values higher than 0.9 and bootstrap values above 50 are shown. Purple bars represent the 95% HPD age intervals at each node. Province and year are next to their accession numbers. Bayesian and maximum-likelihood trees were built under the K2 + G substitution model.

The best evolution model for the CP alignment was K2 + G. The strict molecular clock model plus the Bayesian skyline demographic model was the best fitting molecular clock/tree prior model (Table 1). Phylogenetic analysis using Bayesian inference revealed four monophyletic clades. Group 1 included sequences from Pichincha and Tungurahua. Two isolates from Tungurahua collected in 2018 formed group 2. Moreover, sequences from Tungurahua were present in the three clades where babaco was the host. Group 3 was composed only by the three isolates from chamburo sampled in Azuay. Finally, 28 isolates from Imbabura, Pichincha, Tungurahua, Azuay, and Loja, collected between 2016 and 2021, fell within group 4. In this last group, two lineages were formed, and one of these was composed almost exclusively by isolates from Tungurahua (Figure 1).

The reconstructed epidemic history places the possible origin of the most recent common ancestor (MRCA) of the sampled BabMV isolates in 1958.80 (95% highest posterior density, HPD95% = 1881.43–1999.56). The evolution rate of BabMV was estimated to be 1.21 × 10^−3^ nucleotide substitutions site^−1^ year^−1^ (HPD95% = 2.68 × 10^−4^ nucleotide substitutions site^−1^ year^−1^ −2.32 × 10^−3^ nucleotide substitutions site^−1^ year^−1^). Particularly, the possible origins of the MRCAs of BabMV group 1, group 2, group 3, and group 4, date back to 1972.42, 2015.07, 1967.08, and 1986.27, respectively (Figure 1). 

**Figure 2:**
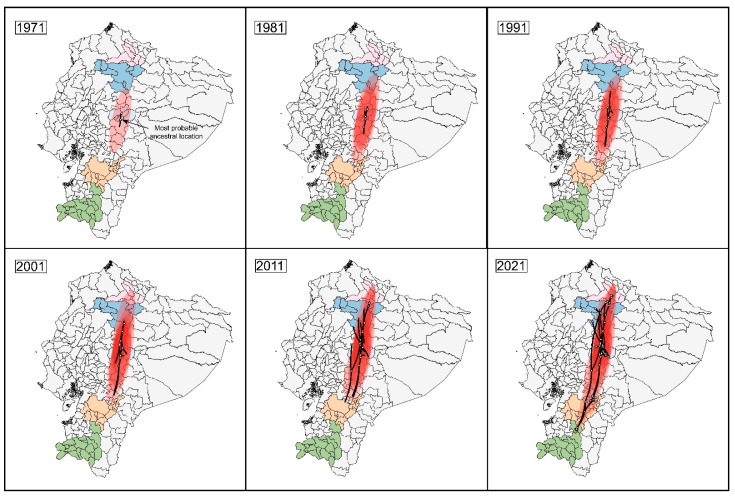
Reconstructed spatiotemporal dynamics of babaco mosaic virus (BabMV) in Ecuador; snapshots showing the viral presence per decade from 1971 to 2021. Black lines show a spatial projection of a representative phylogeny. White circles represent ancestrally estimated geographic locations for nodes in the inferred phylogenetic tree (internal nodes), together with actual locations at tips (external nodes). Colored clouds represent statistical uncertainty in the estimated locations of BabMV lineages (95% HPD intervals). Highland provinces where babaco is grown extensively are colored: pink, Imbabura; blue, Pichincha; light blue, Tungurahua; orange, Azuay; and green, Loja.

The spatiotemporal diffusion processes into the five major babaco-producing provinces showed a homogenous dispersion of BabMV from Tungurahua (location of the most recent common ancestor) to the north and south of the country (Figure 2). 

**Figure 3:**
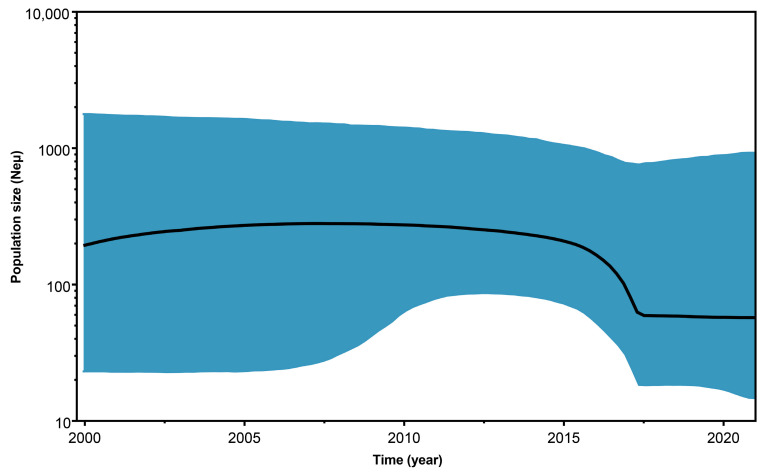
Bayesian skyline plot representing the demographic history of babaco mosaic virus from 2000 to 2021. The black solid line indicates the median population size, and the 95% highest posterior density limits are shown by the blue area. Neμ (effective population size × mutation rate per site per generation).

Reconstruction of the demographic history of BabMV during the period 2000 to 2021, using the Bayesian skyline plot, revealed that the BabMV population size in Ecuador experienced multiple shifts (Figure 3). An increasing and concave downward curve is seen until 2008, and then decreased slowly until mid-2015 when it rapidly decreased until mid-2017. Since then it remained almost constant through 2021.

### 2.3. Three-Dimensional Structure of the BabMV Coat Protein

Indeed, the three-dimensional structures of proteins are highly conserved compared to the DNA sequence and even the protein’s primary structure. For the present case, the molecular differences for the virus lineages will not change the capsid structure significantly. After alignment of the gene under study, the differences between the primary structures corresponded to a few amino acids that would result in little change in the overall structure (Figure S1). Nevertheless, such changes may be significant for molecular recognition considering a single amino acid substitution could change the physicochemical properties at a specific protein surface and therefore change the strength or even the occurrence of an interaction. We present here only the overall structure of the capsid and discuss the relation with other viruses. It is beyond the scope and capabilities of computational methods to evaluate all the possible interactions without studying the possible protein targets of the interaction of the viral protein. Such a study is not within the scope of the current paper.

The capsid structure of potexviruses has been difficult to crystallize due to its intrinsic flexibility; consequently, it was impossible for many years to obtain a high-resolution fiber X-ray diffraction or a cryo-EM structure. An analysis of the normalized B-factor per residue shows a critical number of residues with B-factor values close to or greater than zero. This also agrees with the estimation of disorder protein sequence analysis as the CP has intrinsically disordered regions (score > 0.5) for the C and N terminal regions. In summary, the C and N terminals are the most flexible (Figures S2 and S3), which results in a protein that does not crystallize readily.

**Figure 4:**
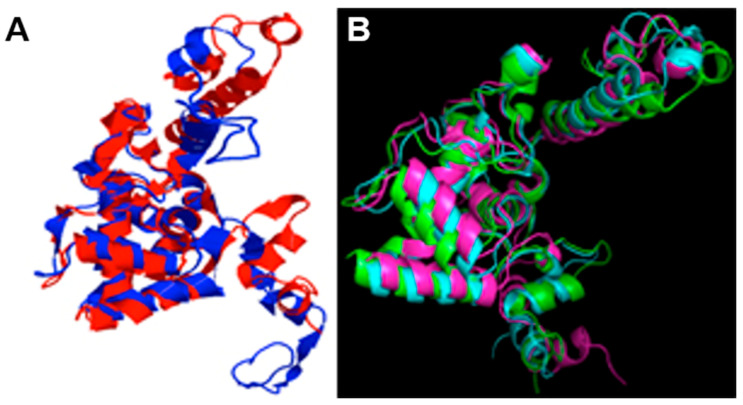
(**A**) Original model of the babaco mosaic virus coat protein (red), built with the I-TASSER homology modeling algorithm, superposed to a representative structure after 50 ns of molecular dynamics simulation (blue). The TM score between both models is 0.67830. (**B**) Superposition of the representative structure of the main clusters of molecular dynamics simulation during 50 ns where the inherent flexibility of the coat protein is visible. Each color represents one of the cluster-representative structures. Only three models are shown.

**Figure 5:**
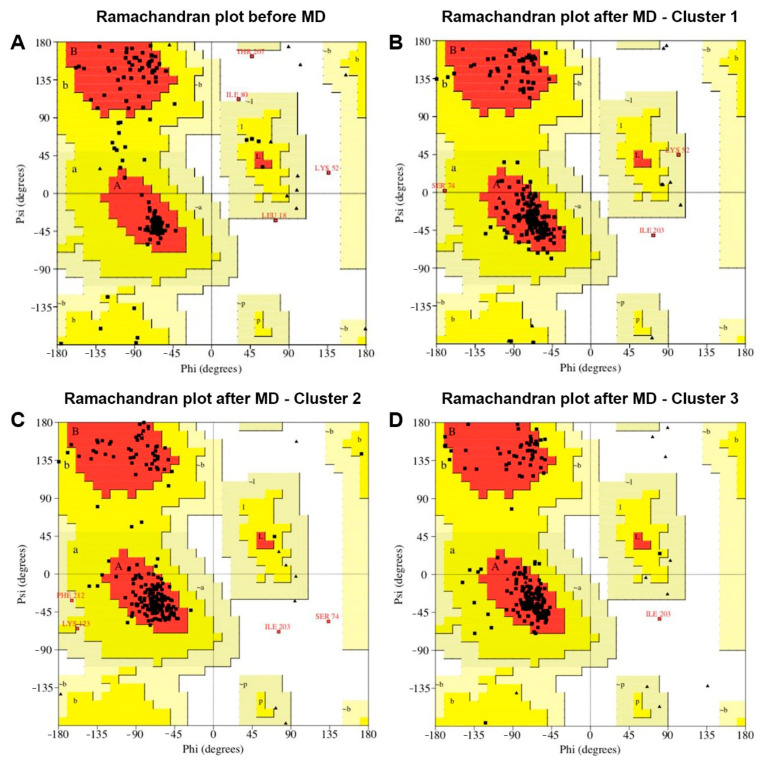
Structure quality assessment using ProCheck for the babaco mosaic virus coat protein with Ramachandran plot analysis. Square dots represent each residue. (**A**) Ramachandran plot of the structure before molecular dynamics refinement. Diagrams (**B**–**D**) show the Ramachandran plots for three different cluster representatives of the molecular dynamics refinement. Upper case letters (A, B, L, P) indicate most favored secondary structures, lower case indicate additional allowed regions (a, b, l, p). After molecular dynamics, most of the residues in the allowed regions move to the core region. The diagram also shows that most residues are in the alpha-helix region (the red region between the top and bottom left quadrants), and some at the beta-sheet region (top left quadrant).

Among the five models generated with I-TASSER, model one had a C-score of 1.52, which indicates high confidence. C-scores are typically in the range (−5, 2). The model with the higher score was chosen for all further refining, analysis, and plots. All models included the molecular dynamics (MD) cluster-representative conformations and were assessed with different molecular structure metrics. In summary, all metrics showed improvement upon refinement with MD simulation. (Tables S1–S3). The original model (Figure 4A, shown in red) was built with the I-TASSER homology modeling algorithm. The Ramachandran plots (Figure 5) show the effect of the MD refining and depict the preponderance of alpha-helix in the structure of the BabMV CP. In addition, Figure 4B shows the original model superposed to a representative structure after 50 ns of MD simulation. The TM score between the two models was 0.67830. The TM scores range within the (0–1) interval, where scores higher than 0.5 can be generally assumed the same fold in SCOP/CATH [13]. These results allowed us to proceed with the MD simulations. We used the root mean square deviation (RMSD) metric to confirm that the molecular dynamics reached equilibrium, ensuring that the configurations obtained were closer to reality [15]. Table 2 shows a summary of the CP metrics compared to simulation results for the RNA-dependent-RNA-polymerase (RDRP), triple gene block 1 (TGB1), and ALKb-domain proteins of BabMV. The root mean square fluctuations (RMSF) for CP were higher than the control of viral proteins, which indicated a higher level of flexibility and motility during the simulation. These results suggest that the C and N terminals are intrinsically disordered protein regions necessary for the CP function.

Clustering for the CP protein–MD production configurations resulted in 64 clusters. All of these clusters allowed us to sample the heterogeneity of conformation in the set of CP protein structures generated by the simulation. As shown in Figure 4A, there were differences in the models before and after carrying out the molecular dynamics. The structural models of CP, before MD, were less compact than the configurations after MD simulation (Figure 4B). In the I-TASSER platform, the structural alignment between the BabMV protein model before and after performing the MD resulted in a TM-score 0.6783 for CP, which shows an optimal alignment. A score equal to one indicates two identical configurations.

Figure 4B shows the superposition of the representative structures for each of the main clusters of molecular dynamics simulation where the inherent flexibility of the capsid protein is visible. In agreement with this visually evident result (the structures do not superimpose perfectly, showing different possible conformations adopted by the protein), the RMSF was calculated per conformation. The RMSF analysis strongly suggests that the CP is the most flexible among the four BabMV proteins analyzed in this study (Table 2), and that this protein contains intrinsically disordered regions. 

A similarity search at the structural level, based on the results obtained by DALI and UniProt, showed that the coat protein of BabMV had structural similarity with the coat proteins of pepino mosaic virus (5FN1) [16], bamboo mosaic virus (5A2T) [17], papaya mosaic virus (4DOX) [18], and watermelon mosaic virus (5ODV) [19]. Using PDBeFold, a structural similarity analysis between the CP of BabMV and bamboo mosaic virus (5A2T) resulted in an alignment quality of 0.43 (1 is the best score), a 20% amino acid sequence identity, and 73% identity for the protein secondary structure.

## 3. Discussion

BabMV is a potexvirus that is widely distributed in the main babaco-producing provinces of Ecuador; it has been previously reported to infect other caricaceaes such as papaya and chamburo [9]. In this work, a total of 36 BabMV isolates were obtained from babaco (*n* = 33) and chamburo (*n* = 3) plants. Whereas the motif KFAAFDFFDGV, highly conserved in the CP of potexviruses [20,21], was found in 35 out of 36 CP sequences analyzed, the variant KFAAFDFFDG**G** was present in one isolate from Pichincha. Nonetheless, the charge and polarity remain in both. Other variants such as KFAGFDFFEGV, KFAGFDFFDGV, KFAAFDFFNGV, RFAAFDFFEGV, RFAAFDFFNGV, KFAAFDFFDAV, and KWAAFDTFDAL have been identified in other members of the genus *Potexvirus* [20]. Although the charge and polarity change in some motif variants, they remain conserved for the majority.

To calculate the evolution rate of the BabMV CP, different combinations of molecular clocks and demographic models were compared through Bayes factors using the marginal likelihood values. The strict clock provided the best fit for our sequence data (Table 1), indicating that the evolutionary rate was the same at every branch in the BabMV phylogenetic tree (Figure 1). Based on the CP gene, this rate was calculated as 1.21 × 10^−3^ nucleotide substitutions site^−1^ year^−1^, which lies within the range reported for other potexviruses [22,23,24]. Traditionally, it was suggested that RNA plant viruses evolve more slowly than RNA animal viruses; however, evidence has shown that certain plant RNA viruses can evolve as rapidly as some animal RNA viruses. Several RNA viruses of the genus *Potyvirus* [25], *Tobamovirus* [26], *Sobemovirus* [27], members of the family *Luteoviridae* [26,28], as well as representatives of the ssDNA genera *Begomovirus* [29,30], *Babuvirus* [31], and *Nanovirus* [32], evolve at rates faster than 10^−5^ substitutions site^−1^ year^−1^ and can reach as high as 10^−2^ or 10^−3^ substitutions site^−1^ year^−1^. The present work supports findings by Gómez et al. (2012) [22] on a distinct potexvirus, pepino mosaic virus, where a high evolutionary rate was also reported. Particularly, the high evolutionary rate of BabMV could have contributed to its hability to emerge in new hosts such as chamburo and to induce symptoms in mechanically inoculated papaya plants [9].

The largest babaco-producing province in Ecuador is Tungurahua, and the highest genetic variability detected in this province agrees with the diversification center of BabMV. During the period from 1982 to 1987, 82% of the total babaco production occurred in Tungurahua [33]; this percentage was reduced to 60% in 2011 and maintained by 2018 with a production of 1841 ton year^−1^ [34]. Isolates from Tungurahua were present in all BabMV groups. Group 4 included isolates from the five studied provinces. The lack of geographic structure in the phylogeny can be explained by the constant exchange of plant-propagating material, e.g., stem cuttings, which are used for asexual reproduction. A common pattern in the babaco production system includes nursery operations in southern provinces, such as Azuay and Loja, and shipping to northern provinces such as Tungurahua and Pichincha for production under greenhouse conditions.

A babaco-infecting potexvirus, tentatively named babaco yellow mosaic virus, was reported from Italy in 1989, but no genome sequence has been provided [11]. According to our findings, the origin of the most common recent ancestor of BabMV was estimated to have occurred in late 1958, and considering the year of the babaco introduction to Italy (1985), we support the notion that the potexvirus reported from Italy is an isolate of BabMV, as previously suggested by Alvarez-Quinto et al. (2017) [9].

During the decade of 1990, Ecuadorian babaco experienced a fresh fruit export boom to Europe and Colombia [33]. However, babaco production was hindered by biotic stress factors, including babaco vascular wilt (BVW) that surged in 1996, spread rapidly, and remains as one of the most important diseases of this plant. BVW could have been dispersed along with others such as BabMV, which population size experienced slow growth from 2000 to 2008 (Figure 3). This behavior correlates well with the limited babaco production in Ecuador at the beginning of the 2000s [33,35,36]. 

The virome of babaco was recently explored by Cornejo-Franco et al. (2020) [10], using high throughput sequencing. Coinfection cases of BabMV with babaco cheravirus-1, babaco cryptic virus 1, babaco nepovirus-1, babaco endogenous pararetrovirus, and the potyvirus papaya ringspot virus were found. To the best of our knowledge, to date PapMV has not been reported to infect babaco plants.

In order to understand the cross-reactivity between PapMV-based antibodies with BabMV [9]—being these two different potexvirus species—we performed an antibody epitope prediction. Figure S4 shows the three-dimensional and sequence alignment between the CPs of PapMV and BabMV. Interestingly, the CPs of these potexviruses share high structural conservation with a RMSD around 1.58 Å for the backbone. Moreover, antibody epitopes prediction based upon the 3D structure of the proteins display common structural epitope region in the zone where proteins align (Tables S4 and S5).

BabMV has a flexible filamentous morphology and, among other viruses that cause diseases to economically important crops, belongs to the family *Alphaflexiviridae* [9]. Structural studies on these viruses have been a challenge due to their inherent flexibility. Parameters regarding the modeling of the BabMV capsid (B-normalized factor, TM-score for the alignment between the structure before and after MD simulation, and DISOPRED probability estimate for each residue to be disordered) point toward the critical flexibility of the BabMV CP. The comparison between the MD simulation (TM-scores for the alignment of the other proteins with the homology model, and RMSF values) for the CP and for the other proteins of the virus also suggests greater flexibility for the CP. The CP fold is the same as previously reported structures for PepMV, BabMV, and PapMV. The essential elements for the self-assembly of the capsid are conserved in the models generated for the BabMV CP. The flexuous nature of the capsid in some viruses challenges its experimental study, but also the algorithms that model the structure from sequence. This difficulty underscores the importance of using an algorithm that uses reported structures (I-TASSER), which are critical to obtain an appropriate folding for in-depth analysis of proteins of recently characterized viruses, such as the CP of BabMV studied here.

The intrinsically disordered protein regions prompted us to analyze their composition and found that they contain charged or polar amino acids and lack hydrophobic amino acids. From the molecular dynamics results, we observed that these regions fluctuate between different conformational states in minimal simulation time lapses. The visual cues from the MD trajectory about the flexibility of these regions were checked quantitatively with the RMSD and RMSF analysis. In the general case, these fluctuations contribute to the function of the proteins by allowing them to interact with different molecules and have been observed in other viruses [37,38]. They bind with minimal affinity but maintain a high level of specificity.

## 4. Materials and Methods

### 4.1. Collection of Virus Isolates, Viral RNA, and Sequencing

**Figure 6:**
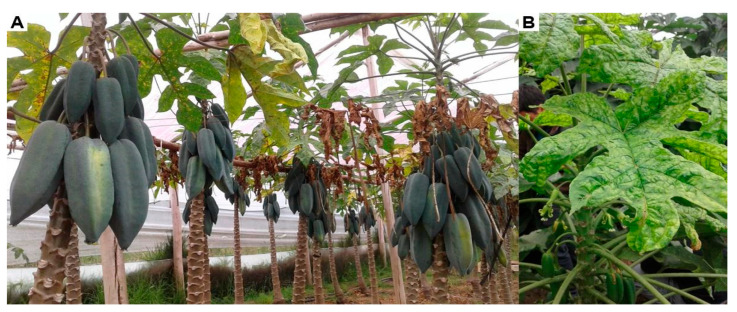
Babaco leaves showing symptoms of chlorosis, mottling, and mosaic, in greenhouse cultivars, Loja, Ecuador. (**A**) Babaco plants. (**B**) A close-up view of a leaf.

Babaco and chamburo leaves showing symptoms of chlorosis, leaf mottling, and mosaic were collected from greenhouses (Figure 6) of the five major babaco-growing provinces of Ecuador: Imbabura, Pichincha, Tungurahua, Azuay, and Loja, between 2016 and 2021. Samples were lyophilized and stored at −20 °C until used. Details of the BabMV isolates used in this study—place of origin, latitude, longitude, host, and GenBank accession number—are shown in Table 3.

Viral RNAs were extracted from 50 mg of symptomatic leaves using the SV Total RNA Isolation System (Promega, Madison, WI, USA). The synthesis of cDNA was carried out using M-MLV Reverse Transcriptase and random primers (Invitrogen, Carlsbad, CA, USA), according to the manufacturer instructions. PCR reactions were performed using GoTaq^®^ Green Master Mix (Promega, Madison, WI, USA) and primers designed in this study to amplify the whole coat protein (CP): virion-sense primer (5′-GCTGTTTTCTTAGTTATCTAG-3′) and complementary-sense primer (5′-GAGGCAAACCTACTCIGG-3′). The PCR mixture contained 200 ng of cDNA, 1U of GoTaq^®^ Green Master Mix, 10 µM of each primer and nuclease-free water to a final reaction volume of 25 µL. PCR cycling conditions consisted of an initial denaturation at 97 °C for 2 min, followed by 35 cycles of denaturation at 95 °C for 20 s, annealing at 42 °C for 30 s and extension at 72 °C for 50 s, and a final extension step at 72 °C for 5 min. 

The PCR products were sequenced in both directions and assembled in Geneious v10.2.3 [39]. Pairwise distances between 36 CP sequences, 35 obtained in this study and one previously reported (GenBank accession number MF978248) [9], were computed using MEGA × [40] to determine, based on species demarcation criteria for the genus *Potexvirus*, whether the sequences belonged to BabMV [14]. 

### 4.2. Recombination Analysis

After aligning the 36 CP nucleotide sequences using MUSCLE [37] in MEGA X, possible recombination events were assessed by using the RDP [41], GENECONV [42], Chimaera [43], MaxChi [44], BootScan [45], SiScan [46], and 3Seq [47] methods, available in the software RDP4 v4.1 [48]. These analyses were performed using default settings and a Bonferroni p-value cutoff of 0.01. Recombination signals detected by at least three methods were considered as credible evidence of recombination.

### 4.3. Phylogenetic Analysis

The phylogeny of BabMV based on 36 CP sequences was built using a Bayesian Markov chain Monte Carlo method, available in the BEAST v.1.10.4 package [49], using the HKY plus gamma distribution and invariant sites model. Evolutionary rates and timescale were calculated under strict and uncorrelated lognormal-relaxed molecular clock conditions. As coalescent tree priors, demographic models of constant population size and Bayesian skyline were tested. The best molecular clock/tree prior model was chosen based on the Bayes factors of the marginal likelihoods estimated by path sampling (PS) and stepping-stone sampling (SS) approaches [50] where the strength of evidence was determined according to Kass and Raftery (1995) [51]. MCMC analysis was run twice for 50 million generations, sampling every 1000 steps. Convergence to the stationary distribution was assessed using effective sample sizes (ESS) in Tracer v1.7 [52], applying a cutoff value of 200. Tree Annotator of the BEAST package was then used to summarize the results in a maximum clade-credibility (MCC) tree with 10% of the sampling discarded as burn-in. The MCC tree was visualized in FigTree v1.4.2 [50]. A maximum likelihood tree was built in MEGA × using the same alignment. Five hundred bootstrap pseudoreplicates were run to obtain the support values of the clades. SPREAD [53] was used to analyze the spatiotemporal dynamics. Finally, the demographic history of BabMV was estimated through a Bayesian skyline plot using Tracer v1.7, with the log file from the phylogenetic reconstruction.

### 4.4. Molecular Modeling of the Babaco Mosaic Virus Coat Protein

A model corresponding to the BabMV CP was built by automated full-length 3D protein structural predictions using the I-TASSER server [54]. This algorithm uses “full-length atomic models constructed by iterative template-based fragment assembly simulations”. Therefore, the structure generated by the algorithm originates from experimental protein structure data. Most protein structures are obtained from electron microscopy or cryoelectron microscopy for viruses. In this case, 5FN1 [16] was obtained by electron cryomicroscopy, while structure 5A2T [17] corresponds to electron microscopy. Therefore, the interaction with RNA is considered indirectly. Further refinement steps do not significantly change the structure but allow the system to relax slightly and show where the protein has areas of higher flexibility. We used structural information from RCSB PDB entries 5FN1A, 5A2TA, and 4DOXA [18] as templates to build the 3D structures. The 5FN1A and 5A2TA templates describe the refined structure of the filamentous flexible pepino mosaic virus (PepMV) and the bamboo mosaic virus (BaMV) coat protein, respectively, whereas 4DOX describes the crystal structure of PapMV coat protein.

The resulting homology model was used to perform a molecular dynamics (MD) simulation to gain understanding on the inherent flexibility of the flexible filamentous plant viruses CP [52,54], and to carry out a three-dimensional alignment with the PapMV CP to predict common antibody epitopes, which could explain why these viruses are serologically indistinguishable. The model quality assessment of the structure beyond the scores automatically generated in the Zhang’s website included ERRAT, ProQ, QMean4, and Procheck servers [55]. Each of these tools evaluates a different characteristic of the model and, together, they provide a general estimation of the quality of the structures [55,56]. A calculation of the estimated probability per residue for the disorder was done with DISOPRED (http://bioinf.cs.ucl.ac.uk/psipred/, accessed on 1 November 2021) [57].

### 4.5. Molecular Dynamics Simulation

MD simulation of the protein was performed using GROMACS 5.1.4 [58] with the Amber-03 force field. The system was set up as a solvated cubic box filled with SPC216 water molecules described by the TIP3P water model. Sodium and chloride ions were added to simulate biological conditions at a concentration of 0.1 M. Periodic boundary conditions and particle-mesh Ewald electrostatics were used. Energy minimization was performed using the steepest descent algorithm and a convergence parameter of less than 10 kJ mol^−1^ nm^−1^. For equilibration dynamics, we ran NVT and NPT ensemble simulations for 500 ps each. V-rescale thermostat at 298 K and Parrinello–Rahman barostat at 1 bar were used [56].

The equilibrated system was subjected to a 50 ns simulation. The molecular dynamic trajectory was analyzed using GROMACS built-in tools, namely, gmx energy, gmx rmsf, and gmx rms. The trajectory clustering was performed with the built-in tool gmx cluster using an RMSD cutoff of 0.25 nm and the gromos algorithm to determine the cluster representatives [59]. Proteins RDRP, TGB1 and Alkb domain (See Table 2) are reported, for comparison reason only, and their three-dimensional models and MD runs were generated following the same protocols described previously using the appropriate templates. Finally, TM-align [60] was used to compare the alignment of the resulting MD structures with the original model before MD.

### 4.6. Three-Dimensional Structural Alignment and Antibody Epitope Prediction

Pairwise structure alignment of the coat proteins of BabMV and PapMV was performed in the protein structure alignment tool from the RCSB PDB (https://www.rcsb.org/, accessed on 22 May 2022) [61], using the TM-align algorithm. Antibody epitope prediction was performed using the structure-based tool ElliPro, which provides linear and discontinuous antibody-predicted epitopes linked with a score defined as a protrusion index (PI) value averaged over epitope residues [62].

## Figures and Tables

**Table 1:** Marginal likelihood score calculated by path sampling and stepping-stone methods for the strict molecular clock and demographic models tested for the Bayesian inference of babaco mosaic virus coat protein phylogeny.

Clock Model	Demographic Model	Path Sampling	2lnBF	Stepping Stone	2lnBF
Strict	Constant size	−3336.8	63.8	−3336.8	63.4
Strict	Exponential growth	−3336.7	63.8	−3336.7	63.2
Strict	Bayesian skyline	−3304.9	N/A	−3305.1	N/A

**Table 2:** Root mean square fluctuation of several babaco mosaic virus proteins from 50 ns production trajectory.

Protein	Min RMSF (Å)	Max RMSF (Å)	Average RMSF (Å) (*n* = 64 Clusters)
CP	0.634	6.328	2.316
RDRP	0.114	1.195	0.327
TGB1	0.505	5.536	2.079
ALKb-domain	0.721	3.692	1.777

**Table 3:** Babaco mosaic virus isolates used in this study.

Isolate	Origin	Latitude–Longitude	Collection Date	Host	Accession Number	Reference
Tandapi	Pichincha	0.414 S 78.799 W	1 March 2016	Babaco	MF978248	Alvarez-Quinto et al. (2017)
IB1-7	Imbabura	0.357 N 78.241 W	12 August 2017	Babaco	MT240490	This study
IB2-7	Imbabura	0.372 N 78.241 W	12 August 2017	Babaco	MT240491	This study
IB3-7	Imbabura	0.359 N 78.217 W	12 August 2017	Babaco	MT240492	This study
IB4-7	Imbabura	0.334 N 78.239 W	12 August 2017	Babaco	MT240493	This study
PB1-7	Pichincha	0.070 S 78.572 W	12 August 2017	Babaco	MT240494	This study
PB2-7	Pichincha	0.216 S 78.412 W	9 March 2017	Babaco	MT240495	This study
PB3-1	Pichincha	0.038 S 78.564 W	15 June 2021	Babaco	OL771197	This study
PB4-1	Pichincha	0.038 S 78.564 W	15 June 2021	Babaco	OL771198	This study
PB5-1	Pichincha	0.038 S 78.564 W	15 June 2021	Babaco	OL771199	This study
PB6-1	Pichincha	0.038 S 78.564 W	15 June 2021	Babaco	OL771200	This study
TB1-7	Tungurahua	1.176 S 78.559 W	17 September 2017	Babaco	MT240496	This study
TB2-7	Tungurahua	1.159 S 78.549 W	17 September 2017	Babaco	MT240497	This study
TB3-7	Tungurahua	1.294 S 78.488 W	17 September 2017	Babaco	MT240498	This study
TB4-7	Tungurahua	1.295 S 78.487 W	17 September 2017	Babaco	MT240499	This study
TB1-8	Tungurahua	1.418 S 78.405 W	24 January 2018	Babaco	MT240500	This study
TB2-8	Tungurahua	1.414 S 78.429 W	24 January 2018	Babaco	MT240501	This study
TB3-8	Tungurahua	1.404 S 78.406 W	24 January 2018	Babaco	MT240502	This study
TB4-8	Tungurahua	1.404 S 78.399 W	24 January 2018	Babaco	MT240503	This study
TB5-8	Tungurahua	1.399 S 78.406 W	24 January 2018	Babaco	MT240504	This study
TB1-1	Tungurahua	1.180 S 78.560 W	13 April 2021	Babaco	MZ490607	This study
TB2-1	Tungurahua	1.180 S 78.560 W	13 April 2021	Babaco	MZ490608	This study
TB3-1	Tungurahua	1.320 S 78.470 W	13 April 2021	Babaco	MZ490609	This study
TB4-1	Tungurahua	1.340 S 78.470 W	13 April 2021	Babaco	MZ490610	This study
TB5-1	Tungurahua	1.330 S 78.470 W	13 April 2021	Babaco	MZ490611	This study
TB6-1	Tungurahua	1.340 S 78.470 W	13 April 2021	Babaco	MZ490612	This study
AB1-7	Azuay	2.727 S 78.779 W	20 November 2017	Babaco	MT240505	This study
AB2-7	Azuay	2.748 S 78.773 W	20 November 2017	Babaco	MT240506	This study
LB1-7	Loja	3.614 S 79.261 W	20 November 2017	Babaco	MT240507	This study
LB2-7	Loja	3.610 S 79.265 W	20 November 2017	Babaco	MT240508	This study
LB3-7	Loja	3.598 S 79.312 W	20 November 2017	Babaco	MT240509	This study
LB4-7	Loja	3.594 S 79.274 W	20 November 2017	Babaco	MT240510	This study
LB5-7	Loja	3.584 S 79.278 W	20 November 2017	Babaco	MT240511	This study
AC1-6	Azuay	2.796 S 78.768 W	15 June 2016	Chamburo	MT240512	This study
AC2-6	Azuay	2.796 S 78.768 W	15 June 2016	Chamburo	MT240513	This study
AC3-6	Azuay	2.796 S 78.768 W	15 June 2016	Chamburo	MT240514	This study

## Data Availability

The coat protein sequences of BabMV isolates found in this study have been deposited in the NCBI database (https://www.ncbi.nlm.nih.gov/genbank/, accessed on 8 December 2021).

## Supplementary Materials

The following are available online at https://www.mdpi.com/article/10.3390/plants11131646/s1, Figure S1: Babaco mosaic virus coat proteins alignment with the overall structure. Figure S2: Normalized B-factor for the babaco mosaic virus coat protein model generated with I-TASSER. Figure S3: A probability estimate of the disorder per residue with DISOPRED for the coat protein of babaco mosaic virus. Both C and N terminals show prevalence of disorder, in this case an indicator of the flexibility of the BabMV CP. Figure S4: Three-dimensional (A) and sequence (B) alignment between the coat proteins of babaco mosaic virus (orange) and papaya mosaic virus (blue). Table S1: Structure quality assessment with ERRAT for the babaco mosaic virus coat protein. Table S2: Structure quality assessment with Verify 3D for the babaco mosaic virus coat protein. Table S3: Structure quality assessment with ProCheck for the babaco mosaic virus coat protein. Ramachandran plot residue analysis. Table S4: Predicted linear epitopes of the coat proteins of BabMV and PapMV. Table S5: Predicted discontinuous epitopes of the coat proteins of BabMV and PapMV.
