# Supplementary material for: Phylodynamics and Coat Protein Analysis of Babaco Mosaic Virus in Ecuador
Source: Plants (Basel). 2022 Jun 22;11(13):1646. doi: 10.3390/plants11131646 (PMC9268947; doi:10.3390/plants11131646)
Supplement: Supplementary file 1 [file plants-11-01646-s001.zip › plants-1730088-supplementary.pdf]

Codon Alignment View

| | | | | |

MF978248|Pichincha|2

ATGAGTGGAAAGTCAGAGTCATCCAACACTGGAAACTCCCCCTTCCCAAATCTCACTAAA 60

_M__S__G__K__S__E__S__S__N__T__G__N__S__P__F__P__N__L__T__K_

MT240490|Imbabura|20

ATGAGTGGAAAATCAGAGTCATCCAACACTGGAAACTCCCCCTTCCCAAGTCTCACTAAA

_M__S__G__K__S__E__S__S__N__T__G__N__S__P__F__P__S__L__T__K_

MT240491|Imbabura|20

ATGAGTGGAAAATCAGAGTCATCCAACACTGGAAACTCCCCCTTCCCAAGTCTCACTAAA

_M__S__G__K__S__E__S__S__N__T__G__N__S__P__F__P__S__L__T__K_

MT240492|Imbabura|20

ATGAGTGGAAAATCAGAGTCATCCAACACTGGAAACTCCCCCTTCCCAAGTCTCACTAAA

_M__S__G__K__S__E__S__S__N__T__G__N__S__P__F__P__S__L__T__K_

MT240493|Imbabura|20

ATGAGTGGAAAATCAGAGTCATCCAACACTGGAAATTCCCCCTTCCCAAGTCTCACTAAA

_M__S__G__K__S__E__S__S__N__T__G__N__S__P__F__P__S__L__T__K_

MT240494|Pichincha|2

ATGAGTGGAAAGTCAGAGTCATCCAACACTGGAAACTCCCCCTTCCCAAATCTCACTAAA

_M__S__G__K__S__E__S__S__N__T__G__N__S__P__F__P__N__L__T__K_

MT240495|Pichincha|2

ATGAGTGGAAAATCAGAGTCATCTAACATTGGAAACTCCCCTTTCCCAAACATCACTAAA

_M__S__G__K__S__E__S__S__N__I__G__N__S__P__F__P__N__I__T__K_

MT240496|Tungurahua|

ATGAGTGGAAAGTCAGAGTCATCCAACACTGGAAACTCCCCCTTCCCAAGCCTCACTAAA

_M__S__G__K__S__E__S__S__N__T__G__N__S__P__F__P__S__L__T__K_

MT240497|Tungurahua|

ATGGGTGAAAAATCAGAGTCATCCAACACTGGAAACCTCCCCTTCCCAAATCTCACCAAA

_M__G__E__K__S__E__S__S__N__T__G__N__L__P__F__P__N__L__T__K_

MT240498|Tungurahua|

ATGAGTGGAAAGTCAGAGTCATCCAACACTGGAAGCTCCCCCTTCCCAAACCTCACTAAA

_M__S__G__K__S__E__S__S__N__T__G__S__S__P__F__P__N__L__T__K_

MT240499|Tungurahua|

ATGAGTGGAAAGTCAGAGTCATCCAACACTGGAAGCTCCCCCTTCCCAAACCTCACTAAA

_M__S__G__K__S__E__S__S__N__T__G__S__S__P__F__P__N__L__T__K_

MT240500|Tungurahua|

ATGAGTGGGAAGTCAGAGTCATCCAACGCTGGGAACTCCCCCTTCCCAAATCTTACCAAA

_M__S__G__K__S__E__S__S__N__A__G__N__S__P__F__P__N__L__T__K_

MT240501|Tungurahua|

ATGAGTGGAAAGTCAGAGTCATCCAACACTGGAAACTCCCCCTTCCCAAATCTCACTAAA

_M__S__G__K__S__E__S__S__N__T__G__N__S__P__F__P__N__L__T__K_

MT240502|Tungurahua|

ATGAGTGGAAAATCAGAGTCATCAAACACTGGAAACTCCCCCTTCCCAAATCTCACCAAA

_M__S__G__K__S__E__S__S__N__T__G__N__S__P__F__P__N__L__T__K_

MT240503|Tungurahua|

ATGAGTGGAAAGTCAGAGTCATCCAACACTGGAAACTCCCCCTTCCCAAATCTCACTAAA

_M__S__G__K__S__E__S__S__N__T__G__N__S__P__F__P__N__L__T__K_

MT240504|Tungurahua|

ATGAGTGGAAAATCAGAGTCATCAAACACTGGAAACTCCCCCTTCCCAAATCTCACCAAA

_M__S__G__K__S__E__S__S__N__T__G__N__S__P__F__P__N__L__T__K_

MT240505|Azuay|2017|

ATGAGTGGAAAATCAGAGTCATCAAACACTGGAAACTCCCCCTTCCCAAATCTCACTAAA

_M__S__G__K__S__E__S__S__N__T__G__N__S__P__F__P__N__L__T__K_

MT240506|Azuay|2017|

ATGAGTGGAAAATCAGAGTCATCAAACACTGGAAACTCCCCCTTCCCAAATCTCACTAAA

_M__S__G__K__S__E__S__S__N__T__G__N__S__P__F__P__N__L__T__K_

MT240507|Loja|2017|B

ATGAGTGGAAAATCAGAGTCATCAAACACTGGAAACTCCCCCTTCCCAAATCTCACTAAA

_M__S__G__K__S__E__S__S__N__T__G__N__S__P__F__P__N__L__T__K_

MT240508|Loja|2017|B

ATGAGTGGAAAATCAGAGTCATCAAACACTGGAAACTCCCCCTTCCCAAATCTCACTAAA

_M__S__G__K__S__E__S__S__N__T__G__N__S__P__F__P__N__L__T__K_

MT240509|Loja|2017|B

ATGAGTGGAAAATCAGAGTCATCAAACACTGGAAACTCCCCCTTCCCAAATCTCACTAAA

_M__S__G__K__S__E__S__S__N__T__G__N__S__P__F__P__N__L__T__K_

MT240510|Loja|2017|B

ATGAGTGGAAAATCAGAGTCATCAAACACTGGAAACTCCCCCTTCCCAAATCTCACTAAA

_M__S__G__K__S__E__S__S__N__T__G__N__S__P__F__P__N__L__T__K_

MT240511|Loja|2017|B

ATGAGTGGAAAATCAGAGTCATCAAACACTGGAAACTCCCCCTTCCCAAATCTCACTAAA

_M__S__G__K__S__E__S__S__N__T__G__N__S__P__F__P__N__L__T__K_

MT240512|Azuay|2016|

ATGAGTGGAAAATCAGAGTCATCCAACACTGGAAACCCCCCTTTCCCAAACCTCACTAAA

_M__S__G__K__S__E__S__S__N__T__G__N__P__P__F__P__N__L__T__K_

MT240513|Azuay|2016|
ATGAGTGGAAAATCAGAGTCATCCAACACTGGAAACCCCCCTTCCCAAACCTCACCAAA
_M__S__G__K__S__E__S__S__N__T__G__N__P__P__F__P__N__L__T__K_
MT240514|Azuay|2016|
ATGAGTGGAAAATCAGAGTCATCCAACATTGGAAACTTCCCCTTCCCAAATCTCACCAAA
_M__S__G__K__S__E__S__S__N__I__G__N__F__P__F__P__N__L__T__K_
MZ490607|Tungurahua|
ATGAGTGGAAAGTCAGAGTCATCCAACACTGGAAACTCCCCCTTCCCAAGCCTCACTAAA
_M__S__G__K__S__E__S__S__N__T__G__N__S__P__F__P__S__L__T__K_
MZ490608|Tungurahua|
ATGAGTGGAAAGTCAGAGTCATCCAACACTGGAAACTCCCCCTTCCCAAGCCTCACTAAA
_M__S__G__K__S__E__S__S__N__T__G__N__S__P__F__P__S__L__T__K_
MZ490609|Tungurahua| -----------
ATCAGAGTCGTCCAACACTGGAAACTCCCCCTTCCCAAATCTCACTAAA
_-__-__-__#__S__E__S__S__N__T__G__N__S__P__F__P__N__L__T__K_
MZ490610|Tungurahua|
ATGAGTGGAAAGTCAGAGTCATCCAACACTGGAAACTCCCCCTTCCCAAACCTCACCAAA
_M__S__G__K__S__E__S__S__N__T__G__N__S__P__F__P__N__L__T__K_
MZ490611|Tungurahua|
ATGAGTGGAAAGTCAGAGTCATCCAACACTGGAAACTCCCCCTTCCCAAACCTCACCAAA
_M__S__G__K__S__E__S__S__N__T__G__N__S__P__F__P__N__L__T__K_
MZ490612|Tungurahua|
ATGAGTGGGAAATCGGAGTCAGCCAACACCGGAAACTCCCCCTTTCCAAATATCACTAAA
_M__S__G__K__S__E__S__A__N__T__G__N__S__P__F__P__N__I__T__K_
OL771197|Pichincha|2
ATGAGTGGAAAATCAGAGTCATCAAACACTGGAAACTTCCCCTTCCCAAATCTCACTAAA
_M__S__G__K__S__E__S__S__N__T__G__N__F__P__F__P__N__L__T__K_
OL771198|Pichincha|2
ATGAGTGGAAAATCAGAGTCATCAAACACTGGAAACTTCCCCTTCCCAAATCTCACTAAA
_M__S__G__K__S__E__S__S__N__T__G__N__F__P__F__P__N__L__T__K_
OL771199|Pichincha|2
ATGAGTGGAAAATCAGAGTCATCCAACATTGGAAACTCCCCCTTCCCAAATCTCACCAAA
_M__S__G__K__S__E__S__S__N__I__G__N__S__P__F__P__N__L__T__K_
OL771200|Pichincha|2
ATGAGTGGAAAATCAGAATCATCCAACATTGGAAACTCCCCTTTTCCAAATATCACTAAA
_M__S__G__K__S__E__S__S__N__I__G__N__S__P__F__P__N__I__T__K_

| | | | | |

MF978248|Pichincha|2

GAGACAATGGCCAGCTTCAACTTCAAACCAAGTTCAAACTTGCTACCTAGCGAGGAAGAG 120

_E__T__M__A__S__F__N__F__K__P__S__S__N__L__L__P__S__E__E__E_

MT240490|Imbabura|20

GAGACAATGGCCAGCTTCAACTTCAAACCAAGTTCAAACTTGCTACCTAGCGAGGAAGAG

_E__T__M__A__S__F__N__F__K__P__S__S__N__L__L__P__S__E__E__E_

MT240491|Imbabura|20

GAAACAATGGCCAGCTTCAACTTCAAACCAAGTTCAAACTTGCTACCTAGCGAGGAAGAG

_E__T__M__A__S__F__N__F__K__P__S__S__N__L__L__P__S__E__E__E_

MT240492|Imbabura|20

GAGACAATGGCCAGCTTCAACTTCAAACCAAGTTCAAACTTGCTACCTAGCGAGGAAGAG

_E__T__M__A__S__F__N__F__K__P__S__S__N__L__L__P__S__E__E__E_

MT240493|Imbabura|20

GAGACAATGGCCAGCTTTAACCTCAAACCAAGTTCAAACTTGCTACCTAGCGAGGAAGAG

_E__T__M__A__S__F__N__L__K__P__S__S__N__L__L__P__S__E__E__E_

MT240494|Pichincha|2

GAGACAATGGCCAACTTCAACTTCAAACCAAGTTCAAACTTGCTGCCCAGCGAGGAAGAG

_E__T__M__A__N__F__N__F__K__P__S__S__N__L__L__P__S__E__E__E_

MT240495|Pichincha|2

GAGACAATGGCCAGCTTTAATTTTAAACCAAGTTCAAATTTGCTGCCTAGCGAGGATGAG

_E__T__M__A__S__F__N__F__K__P__S__S__N__L__L__P__S__E__D__E_

MT240496|Tungurahua|

GAGACGATGGCCAGCTTCAACTTTAAACCAAGTTCAAACTTGCTACCTAGCGAGGAAGAG

_E__T__M__A__S__F__N__F__K__P__S__S__N__L__L__P__S__E__E__E_

MT240497|Tungurahua|

GAGACAATGGCCAGCTTCAACTTCAAACCAAGTTCAAACTTGCTACCTAGCCAGGAAGAG

_E__T__M__A__S__F__N__F__K__P__S__S__N__L__L__P__S__Q__E__E_

MT240498|Tungurahua|

GAGACGATGGCCAGCTTCAACCTCAAACCAAGTTCAAACTTGCTGCCTAGCGAGGAGGAG

_E__T__M__A__S__F__N__L__K__P__S__S__N__L__L__P__S__E__E__E_

MT240499|Tungurahua|

GAGACAATGGCCAGCTTCAACCTCAAACCAAGTTCAAACTTGCTGCCTAGCGAGGAGGAG

_E__T__M__A__S__F__N__L__K__P__S__S__N__L__L__P__S__E__E__E_

MT240500|Tungurahua|

GAGACAATGGCCAGCTTCAACTTCAAACCAAGTTCAAACTTGCTACCTAGCGAGGAAGAG

_E__T__M__A__S__F__N__F__K__P__S__S__N__L__L__P__S__E__E__E_

MT240501|Tungurahua|

GAGACAATGGCCAGCTTCAACTTCAAACCAAGTTCAAACTTGCTACCTAGCGAGGAAGAG

_E__T__M__A__S__F__N__F__K__P__S__S__N__L__L__P__S__E__E__E_

MT240502|Tungurahua|

GAGACAATGGCTAGCTTCAATTTCAAACCGAGTTCAAACTTGCTGCCCAGCGAGGACGAG

_E__T__M__A__S__F__N__F__K__P__S__S__N__L__L__P__S__E__D__E_

MT240503|Tungurahua|

GAGACAATGGCCAACTTCAACTTCAAACCAAGTTCAAACTTGCTACCTAGCGAGGAAGAG

_E__T__M__A__N__F__N__F__K__P__S__S__N__L__L__P__S__E__E__E_

MT240504|Tungurahua|

GAGACAATGGCTAGCTTCAATTTCAAACCGAGTTCAAACTTGCTGCCCAGCGAGGACGAG

_E__T__M__A__S__F__N__F__K__P__S__S__N__L__L__P__S__E__D__E_

MT240505|Azuay|2017|

GAGACAATGGCCAGCTTCAACTTCAAACCAAGTTCAAACTTGCTACCTAGCGAGGAAGAG

_E__T__M__A__S__F__N__F__K__P__S__S__N__L__L__P__S__E__E__E_

MT240506|Azuay|2017|

GAGACAATGGCCAGCTTCAACTTCAAACCAAGTTCAAACTTGCTACCTAGCGAGGAAGAG

_E__T__M__A__S__F__N__F__K__P__S__S__N__L__L__P__S__E__E__E_

MT240507|Loja|2017|B

GAGACAATGGCCAGCTTCAACTTCAAACCAAGTTCAAACTTGCTACCTAGCGAGGAAGAG

_E__T__M__A__S__F__N__F__K__P__S__S__N__L__L__P__S__E__E__E_

MT240508|Loja|2017|B

GAGACAATGGCCAGCTTCAACTTCAAACCAAGTTCAAACTTGCTACCTAGCGAGGAAGAG

_E__T__M__A__S__F__N__F__K__P__S__S__N__L__L__P__S__E__E__E_

MT240509|Loja|2017|B

GAGACAATGGCCAGCTTCAACTTCAAACCAAGTTCAAACTTGCTACCTAGCGAGGAAGAG

_E__T__M__A__S__F__N__F__K__P__S__S__N__L__L__P__S__E__E__E_

MT240510|Loja|2017|B

GAGACAATGGCCAGCTTCAACTTCAAACCAAGTTCAAACTTGCTACCTAGCGAGGAAGAG

_E__T__M__A__S__F__N__F__K__P__S__S__N__L__L__P__S__E__E__E_

MT240511|Loja|2017|B

GAGACAATGGCCAGCTTCAACTTCAAACCAAGTTCAAACTTGCTACCTAGCGAGGAAGAG

_E__T__M__A__S__F__N__F__K__P__S__S__N__L__L__P__S__E__E__E_

MT240512|Azuay|2016|

GAGACAATGGCCGGATTCAATTTCAAACCGAGCTCAAACTTGCTGCCTAGTGAGGACGAG

_E__T__M__A__G__F__N__F__K__P__S__S__N__L__L__P__S__E__D__E_

MT240513|Azuay|2016|

GAGACAATGGCCAGATTCAACTTCAAACCGAGCTCTAACTTGCTGCCCAGCGAGGACGAG

_E__T__M__A__R__F__N__F__K__P__S__S__N__L__L__P__S__E__D__E_

MT240514|Azuay|2016|

GAGACAATGGCCAGCTTCAACTTCAAACCGAGCTCTAACTTGCTGCCCAGCGAGGACGAG

_E__T__M__A__S__F__N__F__K__P__S__S__N__L__L__P__S__E__D__E_

MZ490607|Tungurahua|

GAGACGATGGCCAGCTTCAACTTCAAACCAAGTTCAAACTTGCTACCTAGCGAGGAAGAG

_E__T__M__A__S__F__N__F__K__P__S__S__N__L__L__P__S__E__E__E_

MZ490608|Tungurahua|

GAGACGATGGCCAGCTTCAACTTCAAACCAAGTTCAAACTTGCTACCTAGCGAGGAAGAG

_E__T__M__A__S__F__N__F__K__P__S__S__N__L__L__P__S__E__E__E_

MZ490609|Tungurahua|

GAGACAATGGCCAGCTTCAACTTCAAACCAAGTTCAAACTTGCTACCTAGCGAGGAAGAG

_E__T__M__A__S__F__N__F__K__P__S__S__N__L__L__P__S__E__E__E_

MZ490610|Tungurahua|

GAGACAATGGCCAGTTTCAACTTCAAACCAAGTTCAAACTTGCTACCTAGCGAGGAAGAG

_E__T__M__A__S__F__N__F__K__P__S__S__N__L__L__P__S__E__E__E_

MZ490611|Tungurahua|

GAGACAATGGCCAGTTTCAACTTCAAACCAAGTTCAAACTTGCTACCTAGCGAGGAAGAG

_E__T__M__A__S__F__N__F__K__P__S__S__N__L__L__P__S__E__E__E_

MZ490612|Tungurahua|

GAGACAATGGCCAGCTTCAATTTCAAACCAAGCTCAAACTTGCTGCCTAGCGAGGACGAG

_E__T__M__A__S__F__N__F__K__P__S__S__N__L__L__P__S__E__D__E_

OL771197|Pichincha|2

GAGACAATGGCCAGCTTCAACTTCAAACCAAGTTCAAACTTGCTACCTAGCGAGGAAGAG

_E__T__M__A__S__F__N__F__K__P__S__S__N__L__L__P__S__E__E__E_

OL771198|Pichincha|2

GAGACAATGGCCAGCTTCAACTTCAAACCAAGTTCAAACTTGCTACCTAGCGAGGAAGAG

_E__T__M__A__S__F__N__F__K__P__S__S__N__L__L__P__S__E__E__E_

OL771199|Pichincha|2

GAGACAATGGCCAGCTTTAACTTCAAACCAAGTTCAAACTTGCTACCCAGCGAGGAAGAG

_E__T__M__A__S__F__N__F__K__P__S__S__N__L__L__P__S__E__E__E_

OL771200|Pichincha|2

GAGACAATGGCCAGCTTCAATTTCAAACCAAGTTCAAATCTGCTGCCTAGCGAGGATGAG

_E__T__M__A__S__F__N__F__K__P__S__S__N__L__L__P__S__E__D__E_

| | | | | |

MF978248|Pichincha|2

CTCAAGATCATATCCACATTACTGGTGGCAGCTAAAATACCCAACGCAAGCACCACCATT 180

_L__K__I__I__S__T__L__L__V__A__A__K__I__P__N__A__S__T__T__I_

MT240490|Imbabura|20

CTCAAGATCATATCCACATTAATGGTGGCTGCTAAAATACCGAGCGCGAGCACCACCATT

_L__K__I__I__S__T__L__M__V__A__A__K__I__P__S__A__S__T__T__I_

MT240491|Imbabura|20

CTCAAGATTATATCCACATTACTGATGGCAGCTAAAATACCGAGCGCGAGCACCACCATT

_L__K__I__I__S__T__L__L__M__A__A__K__I__P__S__A__S__T__T__I_

MT240492|Imbabura|20

CTCAAAATCATATCCACATTACTGATGGCAGCTAAAATACCAAGCGCGAGCACCACCATT

_L__K__I__I__S__T__L__L__M__A__A__K__I__P__S__A__S__T__T__I_

MT240493|Imbabura|20

CTCAAGATCATATCCACATTACTGGTGGCAGCTAAAATACCAAGCGCGAGCACCACCATT

_L__K__I__I__S__T__L__L__V__A__A__K__I__P__S__A__S__T__T__I_

MT240494|Pichincha|2

CTCAAGATCATATCCACATTACTAGTTGCAGCTAAAATACCAAGCGCGAGTACCACCATT

_L__K__I__I__S__T__L__L__V__A__A__K__I__P__S__A__S__T__T__I_

MT240495|Pichincha|2

CTCAAGACCATATCCGCACTACTGGTGGCTGCCAAAATACCAAGTGCAAGCACCACGATT

_L__K__T__I__S__A__L__L__V__A__A__K__I__P__S__A__S__T__T__I_

MT240496|Tungurahua|

CTCAAGGTTATATCCACATTACTGGTGGCAGCTAAAATACCAAGCGCGAGCACCACCATT

_L__K__V__I__S__T__L__L__V__A__A__K__I__P__S__A__S__T__T__I_

MT240497|Tungurahua|

CTCAAAACCATATCCACATTACTGGTGGCAGCTAAAATACCGAGCGCAAGCACCACCATT

_L__K__T__I__S__T__L__L__V__A__A__K__I__P__S__A__S__T__T__I_

MT240498|Tungurahua|

CTCAAGATCATATCCTCACTACTGGTGGCAGCCAAAGTACCAAGCGTGAGTACCACCATT

_L__K__I__I__S__S__L__L__V__A__A__K__V__P__S__V__S__T__T__I_

MT240499|Tungurahua|

CTCAAGATCATATCCTCACTACTGGTGGCAGCCAAAGTACCAAGCGTGAGTACCACCATT

_L__K__I__I__S__S__L__L__V__A__A__K__V__P__S__V__S__T__T__I_

MT240500|Tungurahua|

CTCAAGATCATATCCACATTACTGGTGGCAGCCAAAATACCAAGCGCGAGCACCACCATT

_L__K__I__I__S__T__L__L__V__A__A__K__I__P__S__A__S__T__T__I_

MT240501|Tungurahua|

CTCAAGATCATATCCACATTACTGGTAGCAGCTAAAATACCAAGCGCAAGCACCACCATT

_L__K__I__I__S__T__L__L__V__A__A__K__I__P__S__A__S__T__T__I_

MT240502|Tungurahua|

CTTAAGGTTATATCCACATTAATGGTGGCAGCTAAGATACCGAGTGCGAGCACTACCATA

_L__K__V__I__S__T__L__M__V__A__A__K__I__P__S__A__S__T__T__I_

MT240503|Tungurahua|

CTCAAGATCATATCCACATTACTGGTGGCAGCTAAAATACCAAGCGCAAGCACCACCATT

_L__K__I__I__S__T__L__L__V__A__A__K__I__P__S__A__S__T__T__I_

MT240504|Tungurahua|

CTTAAGGTTATATCCACATTAATGGTGGCAGCTAAAATACCGAGTGCGAGCACTACCATA

_L__K__V__I__S__T__L__M__V__A__A__K__I__P__S__A__S__T__T__I_

MT240505|Azuay|2017|

CTCAAGACCATATCCACATTACTGGTGGCAGCAAAAATACCGAGTGGGAGCACCACCATT

_L__K__T__I__S__T__L__L__V__A__A__K__I__P__S__G__S__T__T__I_

MT240506|Azuay|2017|

CTCAAGACCATATCCACATTACTGGTGGCAGCAAAAATACCGAGTGGGAGCACCACCATT

_L__K__T__I__S__T__L__L__V__A__A__K__I__P__S__G__S__T__T__I_

MT240507|Loja|2017|B

CTCAAGACCATATCCACATTACTGGTGGCAGCAAAAATACCGAGTGGGAGCACCACCATT

_L__K__T__I__S__T__L__L__V__A__A__K__I__P__S__G__S__T__T__I_

MT240508|Loja|2017|B

CTCAAGACCATATCCACATTACTGGTGGCAGCAAAAATACCGAGTGGGAGCACCACCATT

_L__K__T__I__S__T__L__L__V__A__A__K__I__P__S__G__S__T__T__I_

MT240509|Loja|2017|B

CTCAAGACCATATCCACATTACTGGTGGCAGCAAAAATACCGAGTGGGAGCACCACCATT

_L__K__T__I__S__T__L__L__V__A__A__K__I__P__S__G__S__T__T__I_

MT240510|Loja|2017|B

CTCAAGACCATATCCACATTACTGGTGGCAGCAAAAATACCGAGTGGGAGCACCACCATT

_L__K__T__I__S__T__L__L__V__A__A__K__I__P__S__G__S__T__T__I_

MT240511|Loja|2017|B

CTCAAGACCATATCCACATTACTGGTGGCAGCAAAAATACCGAGTGGGAGCACCACCATT

_L__K__T__I__S__T__L__L__V__A__A__K__I__P__S__G__S__T__T__I_

MT240512|Azuay|2016|

CTTAAGACCATATCCACACTACTGGTGGCAGCTAAGATACCGAGTACAAGCACCACCATT

_L__K__T__I__S__T__L__L__V__A__A__K__I__P__S__T__S__T__T__I_

MT240513|Azuay|2016|

CTTAAGACCATATCCACATTACTGGTGGCGGCCAAAATACCGAGTGCAAGTACCACCATT

_L__K__T__I__S__T__L__L__V__A__A__K__I__P__S__A__S__T__T__I_

MT240514|Azuay|2016|

CTTAAGACCATATCCACATTACTGGTGGCGGCCAAAATACCGAGTGCAAGTACCACCATT

_L__K__T__I__S__T__L__L__V__A__A__K__I__P__S__A__S__T__T__I_

MZ490607|Tungurahua|

CTCAAGGTTATATCCACATTACTGGTGGCAGCTAAAATACCAAGCGCGAGCACCACCATT

_L__K__V__I__S__T__L__L__V__A__A__K__I__P__S__A__S__T__T__I_

MZ490608|Tungurahua|

CTCAAGGTTATATCCACATTACTGGTGGCAGCTAAAATACCAAGCGCGAGCACCACCATT

_L__K__V__I__S__T__L__L__V__A__A__K__I__P__S__A__S__T__T__I_

MZ490609|Tungurahua|

CTCAAGACCATATCCACATTATTGGTGGCAGCTAAAATACCGAGCGTGAGCACCACCATT

_L__K__T__I__S__T__L__L__V__A__A__K__I__P__S__V__S__T__T__I_

MZ490610|Tungurahua|

CTCAAAACCATATCCACATTACTGGTAGCAGCTAAAATACCAAGCGCGAGCACCACCATT

_L__K__T__I__S__T__L__L__V__A__A__K__I__P__S__A__S__T__T__I_

MZ490611|Tungurahua|

CTCAAAACCATATCCACATTACTGGTAGCAGCTAAAATACCAAGCGCGAGCACCACCATT

_L__K__T__I__S__T__L__L__V__A__A__K__I__P__S__A__S__T__T__I_

MZ490612|Tungurahua|

CTTAGGACCATATCCACATTACTGGTGGCAGCTAAAGTACCGAGTGCAAGTACCACCATT

_L__R__T__I__S__T__L__L__V__A__A__K__V__P__S__A__S__T__T__I_

OL771197|Pichincha|2

CTCAAGACCATATCCACATTACTGGTGGCAGCAAAAATACCGAGTGCGAGCACCACCATT

_L__K__T__I__S__T__L__L__V__A__A__K__I__P__S__A__S__T__T__I_

OL771198|Pichincha|2

CTCAAGACCATATCCACATTACTGGTGGCAGCAAAAATACCGAGTGTGAGCACCACCATT

_L__K__T__I__S__T__L__L__V__A__A__K__I__P__S__V__S__T__T__I_

OL771199|Pichincha|2

CTCAAGACCATATCCACATTACTGGTGGCAGCAAAAATACCGAGCATGAGCACCACCATT

_L__K__T__I__S__T__L__L__V__A__A__K__I__P__S__M__S__T__T__I_

OL771200|Pichincha|2

CTCAAGACCATATCCGCACTACTGGTGGCTGCTAAAATACCAAGTACAAGCACCACCATT

_L__K__T__I__S__A__L__L__V__A__A__K__I__P__S__T__S__T__T__I_

| | | | | |

MF978248|Pichincha|2

GTTGCAATGGACTTGGTCAACTTCTGTTATGACAATGGGTCTAGTGTGTACACGGTGATT 240

_V__A__M__D__L__V__N__F__C__Y__D__N__G__S__S__V__Y__T__V__I_

MT240490|Imbabura|20

GTTGCAATGGACTTGGTCAACTTCTGCTATGACAATGGGTCCAGTGCGTACACGGTGATT

_V__A__M__D__L__V__N__F__C__Y__D__N__G__S__S__A__Y__T__V__I_

MT240491|Imbabura|20
GTTGCAATGGACTTGGTCAACTTCTGCTATGACAATGGGTCCAGTGCGTACACGGTGATT
_V__A__M__D__L__V__N__F__C__Y__D__N__G__S__S__A__Y__T__V__I_
MT240492|Imbabura|20
GTTGCAATGGACTTGGTCAACTTCTGCTATGACAATGGGTCCAGTGCGTACACGGTGATT
_V__A__M__D__L__V__N__F__C__Y__D__N__G__S__S__A__Y__T__V__I_
MT240493|Imbabura|20
GTTGCAATGGACTTGGTCAACTTCTGCTATGACAATGGGTCCAGTGCGTACACGGTGATT
_V__A__M__D__L__V__N__F__C__Y__D__N__G__S__S__A__Y__T__V__I_
MT240494|Pichincha|2
GCTGCAATGGACTTGGTTAACTTCTGCTATGACAATGGGTCCAGTGTGTACACGGTGATT
_A__A__M__D__L__V__N__F__C__Y__D__N__G__S__S__V__Y__T__V__I_
MT240495|Pichincha|2
GCTGCCATGGACTTGGTCAACTTTTGCTATGATAATGGATCAAGTGTGTACACAGCAATC
_A__A__M__D__L__V__N__F__C__Y__D__N__G__S__S__V__Y__T__A__I_
MT240496|Tungurahua|
GCTGCAATGGACTTGGTCAACTTCTGCTATGACAATGGGTCCAGTGTGTACACGGTGATT
_A__A__M__D__L__V__N__F__C__Y__D__N__G__S__S__V__Y__T__V__I_
MT240497|Tungurahua|
GTTGCAATGGACTTGGTCAACTTCTGCTATGACAATGGGTCCAGTGTGTACACGGTGATT
_V__A__M__D__L__V__N__F__C__Y__D__N__G__S__S__V__Y__T__V__I_
MT240498|Tungurahua|
GCTGCAATGGACTTGGTCAACTTCTGCTATGACAATGGGTCCAGTGTGTATACGGTGATT
_A__A__M__D__L__V__N__F__C__Y__D__N__G__S__S__V__Y__T__V__I_
MT240499|Tungurahua|
GCTGCAATGGACTTGGTCAACTTCTGCTATGACAATGGGTCCAGTGTGTATACGGTGATT
_A__A__M__D__L__V__N__F__C__Y__D__N__G__S__S__V__Y__T__V__I_
MT240500|Tungurahua|
GCTGCAATGGACTTGGTCAACTTCTGCTATGACAATGGGTCCAGTGTGTACACGGTGATT
_A__A__M__D__L__V__N__F__C__Y__D__N__G__S__S__V__Y__T__V__I_
MT240501|Tungurahua|
GCTGCAATGGACTTGGTCAACTTCTGCTATGACAATGGGTCCAGTGTGTACACGGTGATT
_A__A__M__D__L__V__N__F__C__Y__D__N__G__S__S__V__Y__T__V__I_
MT240502|Tungurahua|
GTTGCAATGGATTTGGTTAACTTCTGCTATGACAATGGATCCAGCGTGTACACAACAGTT
_V__A__M__D__L__V__N__F__C__Y__D__N__G__S__S__V__Y__T__T__V_
MT240503|Tungurahua|
GCTGCAATGGACTTGGTCAACTTCTGCTATGACAATGGGTCCAGTGTGTACACGGTGATT

_A__A__M__D__L__V__N__F__C__Y__D__N__G__S__S__V__Y__T__V__I_

MT240504|Tungurahua|

GTTGCAATGGATTTGGTTAACTTCTGCTATGACAATGGATCCAGCGTGTACACAACAATT

_V__A__M__D__L__V__N__F__C__Y__D__N__G__S__S__V__Y__T__T__I_

MT240505|Azuay|2017|

GTTGCAATGGACTTGGTTAACTTCTGCTATGACAATGGGTCCAGTGTGTATACGGCGATC

_V__A__M__D__L__V__N__F__C__Y__D__N__G__S__S__V__Y__T__A__I_

MT240506|Azuay|2017|

GTTGCAATGGACTTGGTTAACTTCTGCTATGACAATGGGTCCAGTGTGTATACGGCGATC

_V__A__M__D__L__V__N__F__C__Y__D__N__G__S__S__V__Y__T__A__I_

MT240507|Loja|2017|B

GTTGCAATGGACTTGGTTAACTTCTGCTATGACAATGGGTCCAGTGTGTATACGGCGATC

_V__A__M__D__L__V__N__F__C__Y__D__N__G__S__S__V__Y__T__A__I_

MT240508|Loja|2017|B

GTTGCAATGGACTTGGTCAACTTCTGCTATGACAATGGGTCCAGTGTGTACACGGTGATT

_V__A__M__D__L__V__N__F__C__Y__D__N__G__S__S__V__Y__T__V__I_

MT240509|Loja|2017|B

GTTGCAATGGACTTGGTTAACTTCTGCTATGACAATGGGTCCAGTGTGTATACGGCGATC

_V__A__M__D__L__V__N__F__C__Y__D__N__G__S__S__V__Y__T__A__I_

MT240510|Loja|2017|B

GTTGCAATGGACTTGGTCAACTTCTGCTATGACAATGGGTCCAGTGTGTACACGGTGATT

_V__A__M__D__L__V__N__F__C__Y__D__N__G__S__S__V__Y__T__V__I_

MT240511|Loja|2017|B

GTTGCAATGGACTTGGTTAACTTCTGCTATGACAATGGGTCCAGTGTGTATACGGCGATC

_V__A__M__D__L__V__N__F__C__Y__D__N__G__S__S__V__Y__T__A__I_

MT240512|Azuay|2016|

GCCGCAATGGATTTGGTCAACTTCTGCTATGACAACGGATCCAGTGTGTACACGGTAGTT

_A__A__M__D__L__V__N__F__C__Y__D__N__G__S__S__V__Y__T__V__V_

MT240513|Azuay|2016|

GCTGCAATGGACTTGGTCAACTTCTGCTATGACAATGGGTCCAGTGTGTACACGGTAATT

_A__A__M__D__L__V__N__F__C__Y__D__N__G__S__S__V__Y__T__V__I_

MT240514|Azuay|2016|

GCTGCAATGGACTTGGTCAACTTCTGCTATGACAATGGGTCCAGTGTGTACACGGTAATT

_A__A__M__D__L__V__N__F__C__Y__D__N__G__S__S__V__Y__T__V__I_

MZ490607|Tungurahua|

GCTGCCATGGACTTGGTCAACTTCTGCTATGACAATGGGTCCAGTGTGTACACGGTGATT

_A__A__M__D__L__V__N__F__C__Y__D__N__G__S__S__V__Y__T__V__I_

MZ490608|Tungurahua|
GCTGCCATGGACTTGGTCAACTTCTGCTATGACAATGGGTCCAGTGTGTACACGGTGATT
_A__A__M__D__L__V__N__F__C__Y__D__N__G__S__S__V__Y__T__V__I_
MZ490609|Tungurahua|
GTAGCAATGGACTTGGTCAACTTCTGCTATGACAATGGGTCCAGTGCGTACACGGTGATT
_V__A__M__D__L__V__N__F__C__Y__D__N__G__S__S__A__Y__T__V__I_
MZ490610|Tungurahua|
GCTGCAATGGACTTGGTCAACTTCTGCTATGACAACGGGTCCAGTGTGTACACGGTGATC
_A__A__M__D__L__V__N__F__C__Y__D__N__G__S__S__V__Y__T__V__I_
MZ490611|Tungurahua|
GCTGCAATGGACTTGGTCAACTTCTGCTATGACAACGGGTCCAGTGTGTACACGGTGATC
_A__A__M__D__L__V__N__F__C__Y__D__N__G__S__S__V__Y__T__V__I_
MZ490612|Tungurahua|
GCTGCAATGGACTTGGTCAACTTCTGCTATGACAATGGATCCAGTGTGTACACAGCAATT
_A__A__M__D__L__V__N__F__C__Y__D__N__G__S__S__V__Y__T__A__I_
OL771197|Pichincha|2
GTTGCAATGGACTTGGTCAACTTCTGCTATGACAATGGGTCCAGTGTGTACACGGTGATT
_V__A__M__D__L__V__N__F__C__Y__D__N__G__S__S__V__Y__T__V__I_
OL771198|Pichincha|2
GTTGCAATGGACTTGGTCAACTTCTGCTATGACAATGGGTCCAGTGTGTACACGGTGATT
_V__A__M__D__L__V__N__F__C__Y__D__N__G__S__S__V__Y__T__V__I_
OL771199|Pichincha|2
GTGGCAATGGACTTGGTCAACTTCTGCTATGACAATGGGTCCAGTGCGTACACGGTGATT
_V__A__M__D__L__V__N__F__C__Y__D__N__G__S__S__A__Y__T__V__I_
OL771200|Pichincha|2
GCTGCTATGGATTTGGTCAACTTTTGCTACGATAATGGATCAAGCGTTTACACAGCAATT
_A__A__M__D__L__V__N__F__C__Y__D__N__G__S__S__V__Y__T__A__I_

| | | | | |

MF978248|Pichincha|2
TCTGGTGAATCCTCAATCACTGGCATCACTCTGGCCCAGATTGCAAGTATTGTCAAGGCT 300
_S__G__E__S__S__I__T__G__I__T__L__A__Q__I__A__S__I__V__K__A_
MT240490|Imbabura|20
TCTGGTGAATCCTCAGTCACTGGCGTCACTCTGGCCCAGATTGCAAGCATTGTCAAGGCT
_S__G__E__S__S__V__T__G__V__T__L__A__Q__I__A__S__I__V__K__A_
MT240491|Imbabura|20
TCTGGTGAATCCTCAGTCACTGGCGTCACTCTGGCCCAGATTGCAAGCATTGTCAAGGCT
_S__G__E__S__S__V__T__G__V__T__L__A__Q__I__A__S__I__V__K__A_

MT240492|Imbabura|20

TCTGGTGAATCCTCAGTCACTGGCGTCACTCTGGCCCAGATTGCAAGCATTGTCAAAGCT

_S__G__E__S__S__V__T__G__V__T__L__A__Q__I__A__S__I__V__K__A_

MT240493|Imbabura|20

TCTGGTGAATCCTCAGTCACTGGCGTCACTCTGGCCCAGATTGCAAGTATTGTCAAGGCT

_S__G__E__S__S__V__T__G__V__T__L__A__Q__I__A__S__I__V__K__A_

MT240494|Pichincha|2

TCTGGTGAATCATCAATTACTGGCATCACTCTGGCCCAGATTGCAAGCATTGTTAAGGCT

_S__G__E__S__S__I__T__G__I__T__L__A__Q__I__A__S__I__V__K__A_

MT240495|Pichincha|2

TCCGGTGAATCCTCAGTCGCTGGCGTCACTCTGGCCCAGATTGCAAGCATTGTCAAGGCC

_S__G__E__S__S__V__A__G__V__T__L__A__Q__I__A__S__I__V__K__A_

MT240496|Tungurahua|

TCTGGTGAATCCTCAGTCACTGGCATCACTCTGGCCCAGATTGCAAGCATTGTCAAGGCT

_S__G__E__S__S__V__T__G__I__T__L__A__Q__I__A__S__I__V__K__A_

MT240497|Tungurahua|

TCTGGTGAATCTTCAGTCACTGGCGTCACTCTGGCCCAGGTTGCAAGCATAGTCAAGGCT

_S__G__E__S__S__V__T__G__V__T__L__A__Q__V__A__S__I__V__K__A_

MT240498|Tungurahua|

TCTGGTGAATCCTCAGTCACTGGCATCACTCTGGCCCAGATTGCAAGCATTGTCAAGGCT

_S__G__E__S__S__V__T__G__I__T__L__A__Q__I__A__S__I__V__K__A_

MT240499|Tungurahua|

TCTGGTGAATCCTCAGTCCCTGGCATCACTCTGGCCCAGATTGCAAGCATTGTCAAGGCT

_S__G__E__S__S__V__P__G__I__T__L__A__Q__I__A__S__I__V__K__A_

MT240500|Tungurahua|

TCTGGTGAATCCTCAATCACTGGCATCACTTTGGCCCAGATTGCAAGCATTGTCAAAGCT

_S__G__E__S__S__I__T__G__I__T__L__A__Q__I__A__S__I__V__K__A_

MT240501|Tungurahua|

TCTGGTGAATCCTCAATCACTGGCATCACTCTGGCCCAGATTGCAAGCATTGTCAAGGCT

_S__G__E__S__S__I__T__G__I__T__L__A__Q__I__A__S__I__V__K__A_

MT240502|Tungurahua|

TCGGGTGAATCCTCAGTCCCAGGCGTCACTCTGGCCCAGATTGCAAGCATTGTTAAGGCT

_S__G__E__S__S__V__P__G__V__T__L__A__Q__I__A__S__I__V__K__A_

MT240503|Tungurahua|

TCTGGTGAATCCTCAATCACTGGCATCACTCTGGCCCAGATTGCAAGCATTGTCAAGGCT

_S__G__E__S__S__I__T__G__I__T__L__A__Q__I__A__S__I__V__K__A_

MT240504|Tungurahua|

TCGGGTGAATCCTCAGTCACAGGCGTCACTCTGGCCCAGATTGCAAGCATTGTTAAGGCT

_S__G__E__S__S__V__T__G__V__T__L__A__Q__I__A__S__I__V__K__A_

MT240505|Azuay|2017|

TCTGGTGAATCCTCAATCGCTGGCGTCACTCTGGCCCAGATTGCAAGCATTGTCAAGGCC

_S__G__E__S__S__I__A__G__V__T__L__A__Q__I__A__S__I__V__K__A_

MT240506|Azuay|2017|

TCTGGTGAATCCTCAATCGCTGGCGTCACTCTGGCCCAGATTGCAAGCATTGTCAAGGCC

_S__G__E__S__S__I__A__G__V__T__L__A__Q__I__A__S__I__V__K__A_

MT240507|Loja|2017|B

TCTGGTGAATCCTCAATCGCTGGCGTCACTCTGGCCCAGATTGCAAGCATTGTCAAGGCC

_S__G__E__S__S__I__A__G__V__T__L__A__Q__I__A__S__I__V__K__A_

MT240508|Loja|2017|B

TCTGGTGAATCCTCAATCGCTGGCGTCACTCTGGCCCAGATTGCAAGCATTGTCAAGGCC

_S__G__E__S__S__I__A__G__V__T__L__A__Q__I__A__S__I__V__K__A_

MT240509|Loja|2017|B

TCTGGTGAATCCTCAATCGCTGGCGTCACTCTGGCCCAGATTGCAAGCATTGTCAAGGCC

_S__G__E__S__S__I__A__G__V__T__L__A__Q__I__A__S__I__V__K__A_

MT240510|Loja|2017|B

TCTGGTGAATCCTCAATCGCTGGCGTCACTCTGGCCCAGATTGCAAGCATTGTCAAGGCC

_S__G__E__S__S__I__A__G__V__T__L__A__Q__I__A__S__I__V__K__A_

MT240511|Loja|2017|B

TCTGGTGAATCCTCAATCGCTGGCGTCACTCTGGCCCAGATTGCAAGCATTGTCAAGGCC

_S__G__E__S__S__I__A__G__V__T__L__A__Q__I__A__S__I__V__K__A_

MT240512|Azuay|2016|

TCCGGTGAATCCTCAGTTGCTGGCGTCACTTTGGCCCAAATTGCAAGCATTGTCAAGGCT

_S__G__E__S__S__V__A__G__V__T__L__A__Q__I__A__S__I__V__K__A_

MT240513|Azuay|2016|

TCCGGAGAATCTTCAGTTACCGGCATCACCCTGGCTCAGATTGCTAGCATTGTCAAGGCT

_S__G__E__S__S__V__T__G__I__T__L__A__Q__I__A__S__I__V__K__A_

MT240514|Azuay|2016|

TCCGGAGAATCTTCAGTTACCGGCATCACCCTGGCTCAGATTGCTAGCATTGTCAAGGCT

_S__G__E__S__S__V__T__G__I__T__L__A__Q__I__A__S__I__V__K__A_

MZ490607|Tungurahua|

TCTGGTGAATCCTCAGTCACTGGCATCACTCTGGCCCAGATTGCAAGCATTGTCAAGGCT

_S__G__E__S__S__V__T__G__I__T__L__A__Q__I__A__S__I__V__K__A_

MZ490608|Tungurahua|

TCTGGTGAATCCTCAGTCACTGGCATCACTCTGGCCCAGATTGCAAGCATTGTCAAGGCT

_S__G__E__S__S__V__T__G__I__T__L__A__Q__I__A__S__I__V__K__A_

MZ490609|Tungurahua|
TCTGGCGAATCCTCAGTCACTGGCGTCACTCTGGCCCAGATTGCAAGCATTGTCAAGGCT
_S__G__E__S__S__V__T__G__V__T__L__A__Q__I__A__S__I__V__K__A_
MZ490610|Tungurahua|
TCTGGTGAATCCTCAATCACTGGCATCACTCTGGCCCAGATTGCAAGCATTGTCAAGGCT
_S__G__E__S__S__I__T__G__I__T__L__A__Q__I__A__S__I__V__K__A_
MZ490611|Tungurahua|
TCTGGTGAATCCTCAATCACTGGCATCACTCTGGCCCAGATTGCAAGCATTGTCAAGGCT
_S__G__E__S__S__I__T__G__I__T__L__A__Q__I__A__S__I__V__K__A_
MZ490612|Tungurahua|
TCTGGTGAATCCTCAGTCACTGGCGTTACTCTAGCCCAGATTGCAAGCATTGTCAAGGCT
_S__G__E__S__S__V__T__G__V__T__L__A__Q__I__A__S__I__V__K__A_
OL771197|Pichincha|2
TCTGGTGAATCCTCAATCGCTGGCGTCACTCTGGCCCAGATTGCAAGCATTGTCAAGGCC
_S__G__E__S__S__I__A__G__V__T__L__A__Q__I__A__S__I__V__K__A_
OL771198|Pichincha|2
TCTGGTGAATCCTCAATCGCTGGCGTCACTCTGGCCCAGATTGCAAGCATTGTCAAGGCC
_S__G__E__S__S__I__A__G__V__T__L__A__Q__I__A__S__I__V__K__A_
OL771199|Pichincha|2
TCTGGTGAATCCTCAGTCACTGGCGTCACTCTGGCCCAGATTGCAAGTATTGTCAAGGCT
_S__G__E__S__S__V__T__G__V__T__L__A__Q__I__A__S__I__V__K__A_
OL771200|Pichincha|2
TCTGGTGAAAGCTCAGTCGCTGGCGTTACCCTGGCCCAGATTGCAAGCATTGTCAAGGCC
_S__G__E__S__S__V__A__G__V__T__L__A__Q__I__A__S__I__V__K__A_

| | | | | |

MF978248|Pichincha|2
TCCGGCACTTCACTACGAAAATTCTGCCGATTCTTTGCACCAGTAATTTGGAACCTGAGG 360
_S__G__T__S__L__R__K__F__C__R__F__F__A__P__V__I__W__N__L__R_
MT240490|Imbabura|20
TCCGGCACCTCACTACGAAAATTCTGCCGATTCTTTGCACCAGTAATTTGGAACCTGAGG
_S__G__T__S__L__R__K__F__C__R__F__F__A__P__V__I__W__N__L__R_
MT240491|Imbabura|20
TCCGGCACCTCACTACGAAAATTCTGCCGATTCTTTGCACCAGTAATTTGGAACCTGAGG
_S__G__T__S__L__R__K__F__C__R__F__F__A__P__V__I__W__N__L__R_
MT240492|Imbabura|20
TCCGGCACCTCACTACGAAAATTCTGCCGATTCTTTGCACCAGTAATTTGGAACCTGAGG
_S__G__T__S__L__R__K__F__C__R__F__F__A__P__V__I__W__N__L__R_

MT240493|Imbabura|20

TCCGGCACCTCACTACGAAAATTCTGCCGATTCTTTGCACCAGTAATTTGGAACCTGAGG

_S__G__T__S__L__R__K__F__C__R__F__F__A__P__V__I__W__N__L__R_

MT240494|Pichincha|2

TCCGGCACTTCACTACGAAAATTCTGCCGATTCTTTGCACCAGTAATTTGGAACCTGAGA

_S__G__T__S__L__R__K__F__C__R__F__F__A__P__V__I__W__N__L__R_

MT240495|Pichincha|2

TCCGGCACCTCACTGCGAAAATTCTGTCGATTCTTTGCACCAGTGATTTGGAACTTGAGG

_S__G__T__S__L__R__K__F__C__R__F__F__A__P__V__I__W__N__L__R_

MT240496|Tungurahua|

TCCGGCACTTCACTGCGAAAATTCTGCCGATTCTTTGCACCAGTAATTTGGAACCTGAGG

_S__G__T__S__L__R__K__F__C__R__F__F__A__P__V__I__W__N__L__R_

MT240497|Tungurahua|

TCCGGAACCTCACTACGAAAATTCTGCCGATTCTTTGCACCAGTAATTTGGAACCTGAGG

_S__G__T__S__L__R__K__F__C__R__F__F__A__P__V__I__W__N__L__R_

MT240498|Tungurahua|

TCCGGCACTTCACTACGAAAATTCTGCCGATTCTTTGCACCAGTAATTTGGAACCTGAGG

_S__G__T__S__L__R__K__F__C__R__F__F__A__P__V__I__W__N__L__R_

MT240499|Tungurahua|

TCCGGCACTTCACTACGAAAATTCTGCCGATTCTTTGCACCAGTAATTTGGAACCTGAGG

_S__G__T__S__L__R__K__F__C__R__F__F__A__P__V__I__W__N__L__R_

MT240500|Tungurahua|

TCCGGCACTTCACTACGAAAATTCTGCCGATTCTTTGCACCAGTGATTTGGAACCTGAGG

_S__G__T__S__L__R__K__F__C__R__F__F__A__P__V__I__W__N__L__R_

MT240501|Tungurahua|

TCCGGCACTTCACTACGAAAATTCTGCCGATTCTTTGCACCAGTGATTTGGAACCTGAGG

_S__G__T__S__L__R__K__F__C__R__F__F__A__P__V__I__W__N__L__R_

MT240502|Tungurahua|

TCCGGCACCTCACTACGAAAATTTTGCCGATTCTTTGCACCAGTGATTTGGAATCTGAGA

_S__G__T__S__L__R__K__F__C__R__F__F__A__P__V__I__W__N__L__R_

MT240503|Tungurahua|

TCCGGCACTTCACTACGAAAATTCTGCCGATTCTTTGCACCAGTAATTTGGAACCTGAGG

_S__G__T__S__L__R__K__F__C__R__F__F__A__P__V__I__W__N__L__R_

MT240504|Tungurahua|

TCCGGCACCTCACTACGAAAATTTTGCCGATTCTTTGCACCAGTGATTTGGAATCTGAGA

_S__G__T__S__L__R__K__F__C__R__F__F__A__P__V__I__W__N__L__R_

MT240505|Azuay|2017|

TCCGGCACCTCACTACGAAAATTCTGCCGATTCTTTGCACCGGTAATTTGGAACCTGAGA

_S__G__T__S__L__R__K__F__C__R__F__F__A__P__V__I__W__N__L__R_

MT240506|Azuay|2017|

TCCGGCACCTCACTACGAAAATTCTGCCGATTCTTTGCACCGGTAATTTGGAACCTGAGA

_S__G__T__S__L__R__K__F__C__R__F__F__A__P__V__I__W__N__L__R_

MT240507|Loja|2017|B

TCCGGCACCTCACTACGAAAATTCTGCCGATTCTTTGCACCGGTAATTTGGAACCTGAGA

_S__G__T__S__L__R__K__F__C__R__F__F__A__P__V__I__W__N__L__R_

MT240508|Loja|2017|B

TCTGGCACCTCACTACGAAAATTCTGCCGATTCTTTGCACCGGTAATTTGGAACCTGAGA

_S__G__T__S__L__R__K__F__C__R__F__F__A__P__V__I__W__N__L__R_

MT240509|Loja|2017|B

TCCGGCACCTCACTACGAAAATTCTGCCGATTCTTTGCACCGGTAATTTGGAACCTGAGA

_S__G__T__S__L__R__K__F__C__R__F__F__A__P__V__I__W__N__L__R_

MT240510|Loja|2017|B

TCTGGCACCTCACTACGAAAATTCTGCCGATTCTTTGCACCGGTAATTTGGAACCTGAGA

_S__G__T__S__L__R__K__F__C__R__F__F__A__P__V__I__W__N__L__R_

MT240511|Loja|2017|B

TCCGGCACCTCACTACGAAAATTCTGCCGATTCTTTGCACCGGTAATTTGGAACCTGAGA

_S__G__T__S__L__R__K__F__C__R__F__F__A__P__V__I__W__N__L__R_

MT240512|Azuay|2016|

TCCGGCACCTCACTGCGAAAATTCTGCCGATTCTTTGCACCAGTGATCTGGAACCTGAGG

_S__G__T__S__L__R__K__F__C__R__F__F__A__P__V__I__W__N__L__R_

MT240513|Azuay|2016|

TCAGGCACCTCATTACGAAAATTCTGCCGATTCTTTGCACCAGTGATCTGGAACCTGAGG

_S__G__T__S__L__R__K__F__C__R__F__F__A__P__V__I__W__N__L__R_

MT240514|Azuay|2016|

TCAGGCACCTCACTACGAAAATTCTGCCGATTCTTTGCACCAGTGATTTGGAACCTGAGG

_S__G__T__S__L__R__K__F__C__R__F__F__A__P__V__I__W__N__L__R_

MZ490607|Tungurahua|

TCCGGCACTTCACTGCGAAAATTCTGCCGATTCTTTGCACCAGTAATTTGGAACCTGAGG

_S__G__T__S__L__R__K__F__C__R__F__F__A__P__V__I__W__N__L__R_

MZ490608|Tungurahua|

TCCGGCACTTCACTGCGAAAATTCTGCCGATTCTTTGCACCAGTAATTTGGAACCTGAGG

_S__G__T__S__L__R__K__F__C__R__F__F__A__P__V__I__W__N__L__R_

MZ490609|Tungurahua|

TCCGGCACCTCACTACGAAAATTCTGCCGATTCTTTGCACCAGTAATTTGGAACCTGAGA

_S__G__T__S__L__R__K__F__C__R__F__F__A__P__V__I__W__N__L__R_

MZ490610|Tungurahua|

TCCGGCACTTCGCTACGAAAATTTTGCCGATTCTTTGCACCAGTAATTTGGAACCTGAGG

_S__G__T__S__L__R__K__F__C__R__F__F__A__P__V__I__W__N__L__R_

MZ490611|Tungurahua|

TCCGGCACTTCGCTACGAAAATTTTGCCGATTCTTTGCACCAGTAATTTGGAACCTGAGG

_S__G__T__S__L__R__K__F__C__R__F__F__A__P__V__I__W__N__L__R_

MZ490612|Tungurahua|

TCTGGCACCTCACTACGAAAATTCTGCCGATTCTTCGCACCAGTGATCTGGAACCTGAGG

_S__G__T__S__L__R__K__F__C__R__F__F__A__P__V__I__W__N__L__R_

OL771197|Pichincha|2

TCCGGCACCTCACTACGAAAATTCTGCCGATTCTTCGCACCGGTAATTTGGAACCTGAGA

_S__G__T__S__L__R__K__F__C__R__F__F__A__P__V__I__W__N__L__R_

OL771198|Pichincha|2

TCCGGCACCTCACTACGAAAATTCTGCCGATTCTTCGCACCGGTAATTTGGAACCTGAGA

_S__G__T__S__L__R__K__F__C__R__F__F__A__P__V__I__W__N__L__R_

OL771199|Pichincha|2

TCCGGCACCTCACTACGAAAATTCTGCCGATTCTTTGCACCAGTAATTTGGAACCTGAGG

_S__G__T__S__L__R__K__F__C__R__F__F__A__P__V__I__W__N__L__R_

OL771200|Pichincha|2

TCCGGCACTTCACTGCGAAAATTCTGTCGATTCTTTGCACCAGTGATTTGGAACTTGAGG

_S__G__T__S__L__R__K__F__C__R__F__F__A__P__V__I__W__N__L__R_

| | | | | |

MF978248|Pichincha|2

ACTGACAAAGTACCCCCCGCAAACTGGGAGAGCGCTGGGTACAAACCAACTGAGAAATTC 420

_T__D__K__V__P__P__A__N__W__E__S__A__G__Y__K__P__T__E__K__F_

MT240490|Imbabura|20

ACTGACAAAGTACCCCCCGCAAACTGGGAGAGTGCTGGGTTCAAACCAACTGAGAAATTC

_T__D__K__V__P__P__A__N__W__E__S__A__G__F__K__P__T__E__K__F_

MT240491|Imbabura|20

ACTGACAAAGTACCCCCCGCAAACTGGGAGAGTGCTGGGTTCAAACCAACTGAGAAATTC

_T__D__K__V__P__P__A__N__W__E__S__A__G__F__K__P__T__E__K__F_

MT240492|Imbabura|20

ACTGACAAAGTACCCCCCGCAAACTGGGAGAGTGCTGGGTTCAAACCAACTGAGAAATTC

_T__D__K__V__P__P__A__N__W__E__S__A__G__F__K__P__T__E__K__F_

MT240493|Imbabura|20

ACTGACAAAGTACCCCCCGCAAACTGGGAGAGTGCTGGGTTCAAACCAACTGAGAAATTC

_T__D__K__V__P__P__A__N__W__E__S__A__G__F__K__P__T__E__K__F_

MT240494|Pichincha|2

ACTGACAAAGTACCCCCCGCAAACTGGGAGAGTGCTGGGTATAAACCAACTGAGAAATTC

_T__D__K__V__P__P__A__N__W__E__S__A__G__Y__K__P__T__E__K__F_

MT240495|Pichincha|2

ACTGATAAAGTGCCCCCCGCAAACTGGGAGAGTGCTGGGTACAAACCAACTGAAAAATTC

_T__D__K__V__P__P__A__N__W__E__S__A__G__Y__K__P__T__E__K__F_

MT240496|Tungurahua|

ACTGACAAAGTACCCCCCGCAAACTGGGAGAGTGCTGGGTACAAACCAACTGAGAAATTC

_T__D__K__V__P__P__A__N__W__E__S__A__G__Y__K__P__T__E__K__F_

MT240497|Tungurahua|

ACTGACAAAGTACCCCCCGCAAACTGGGAGAGTGCTGGGTTTAAACCAAATGAGAAATTC

_T__D__K__V__P__P__A__N__W__E__S__A__G__F__K__P__N__E__K__F_

MT240498|Tungurahua|

ACTGACAAAGTACCCCCCGCAAACTGGGAGAGTGCTGGGTACAAACCAACTGAAAAATTC

_T__D__K__V__P__P__A__N__W__E__S__A__G__Y__K__P__T__E__K__F_

MT240499|Tungurahua|

ACTGACAAAGTACCCCCCGCAAACTGGGAGAGTGCTGGGTACAAACCAACTGAAAAATTC

_T__D__K__V__P__P__A__N__W__E__S__A__G__Y__K__P__T__E__K__F_

MT240500|Tungurahua|

ACTGACAAAGTACCCCCCGCAAACTGGGAGAGTGCTGGGTACAAACCAACTGAGAAATTC

_T__D__K__V__P__P__A__N__W__E__S__A__G__Y__K__P__T__E__K__F_

MT240501|Tungurahua|

ACTGACAAAGTACCCCCCGCAAACTGGGAGAGTGCTGGGTATAAACCAACTGAGAAATTC

_T__D__K__V__P__P__A__N__W__E__S__A__G__Y__K__P__T__E__K__F_

MT240502|Tungurahua|

ACTGACAAAGTGCCCCCCGCAAACTGGGAAAGTGCTGGGTACAAACCAACTGAGAAATTC

_T__D__K__V__P__P__A__N__W__E__S__A__G__Y__K__P__T__E__K__F_

MT240503|Tungurahua|

ACTGACAAAGTACCCCCCGCAAACTGGGAGAGTGCTGGGTACAAACCAACTGAGAAATTC

_T__D__K__V__P__P__A__N__W__E__S__A__G__Y__K__P__T__E__K__F_

MT240504|Tungurahua|

ACTGACAAAGTGCCCCCCGCAAACTGGGAAAGTGCTGGGTACAAACCAACTGAGAAATTC

_T__D__K__V__P__P__A__N__W__E__S__A__G__Y__K__P__T__E__K__F_

MT240505|Azuay|2017|

ACTGACAAAGTACCCCCCGCAAACTGGGAGAGTGCTGGGTTTAAACCAACTGAGAAATTC

_T__D__K__V__P__P__A__N__W__E__S__A__G__F__K__P__T__E__K__F_

MT240506|Azuay|2017|

ACTGACAAAGTACCCCCCGCAAACTGGGAGAGTGCTGGGTTTAAACCAACTGAGAAATTC

_T__D__K__V__P__P__A__N__W__E__S__A__G__F__K__P__T__E__K__F_

MT240507|Loja|2017|B

ACTGACAAAGTACCCCCCGCAAACTGGGAGAGTGCTGGGTTTAAACCAACTGAGAAATTC

_T__D__K__V__P__P__A__N__W__E__S__A__G__F__K__P__T__E__K__F_

MT240508|Loja|2017|B

ACTGACAAAGTACCCCCCGCAAACTGGGAGAGTGCTGGGTTTAAACCAACTGAGAAATTC

_T__D__K__V__P__P__A__N__W__E__S__A__G__F__K__P__T__E__K__F_

MT240509|Loja|2017|B

ACTGACAAAGTACCCCCCGCAAACTGGGAGAGTGCTGGGTTTAAACCAACTGAGAAATTC

_T__D__K__V__P__P__A__N__W__E__S__A__G__F__K__P__T__E__K__F_

MT240510|Loja|2017|B

ACTGACAAAGTACCCCCCGCAAACTGGGAGAGTGCTGGGTTTAAACCAACTGAGAAATTC

_T__D__K__V__P__P__A__N__W__E__S__A__G__F__K__P__T__E__K__F_

MT240511|Loja|2017|B

ACTGACAAAGTACCCCCCGCAAACTGGGAGAGTGCTGGGTTTAAACCAACTGAGAAATTC

_T__D__K__V__P__P__A__N__W__E__S__A__G__F__K__P__T__E__K__F_

MT240512|Azuay|2016|

ACTGACAAAATACCCCCCGCGAACTGGGAGAGTGCTGGGTACAAACCAACTGAAAAATTC

_T__D__K__I__P__P__A__N__W__E__S__A__G__Y__K__P__T__E__K__F_

MT240513|Azuay|2016|

ACTGACAAAGTGCCCCCCGCCAACTGGGAGAGTGCTGGGTACAAACCAACCGAAAAATTC

_T__D__K__V__P__P__A__N__W__E__S__A__G__Y__K__P__T__E__K__F_

MT240514|Azuay|2016|

ACTGACAAAGTGCCCCCCGCCAACTGGGAGAGTGCTGGGTACAAACCAACCGAGAAATTC

_T__D__K__V__P__P__A__N__W__E__S__A__G__Y__K__P__T__E__K__F_

MZ490607|Tungurahua|

ACTGACAAAGTACCCCCCGCAAACTGGGAGAGTGCTGGGTACAAACCAACTGAGAAATTC

_T__D__K__V__P__P__A__N__W__E__S__A__G__Y__K__P__T__E__K__F_

MZ490608|Tungurahua|

ACTGACAAAGTACCCCCCGCAAACTGGGAGAGTGCTGGGTACAAACCGACTGAGAAATTC

_T__D__K__V__P__P__A__N__W__E__S__A__G__Y__K__P__T__E__K__F_

MZ490609|Tungurahua|

ACTGACAAAGTACCCCCCGCAAACTGGGAGAGTGCTGGGTTCAAACCAAGTGAGAAATTC

_T__D__K__V__P__P__A__N__W__E__S__A__G__F__K__P__S__E__K__F_

MZ490610|Tungurahua|

ACTGACAAAGTACCCCCCGCAAACTGGGAGAGTGCTGGGTACAAACCAACTGAGAAATTT

_T__D__K__V__P__P__A__N__W__E__S__A__G__Y__K__P__T__E__K__F_

MZ490611|Tungurahua|
ACTGACAAAGTACCCCCCGCAAACTGGGAGAGTGCTGGGTACAAACCAACTGAGAAATTT
_T__D__K__V__P__P__A__N__W__E__S__A__G__Y__K__P__T__E__K__F_
MZ490612|Tungurahua|
ACTGACAAAGTACCCCCCGCAAACTGGGAGAGTGCTGGTTACAAACCAACTGAAAAGTTC
_T__D__K__V__P__P__A__N__W__E__S__A__G__Y__K__P__T__E__K__F_
OL771197|Pichincha|2
ACTGACAAAGTACCCCCCGCAAACTGGGAGAGTGCTGGGTTTAAACCAACTGAGAAATTC
_T__D__K__V__P__P__A__N__W__E__S__A__G__F__K__P__T__E__K__F_
OL771198|Pichincha|2
ACTGACAAAGTACCCCCCGCAAACTGGGAGAGTGCTGGGTTTAAACCAACTGAGAAATTC
_T__D__K__V__P__P__A__N__W__E__S__A__G__F__K__P__T__E__K__F_
OL771199|Pichincha|2
ACTGACAAAGTACCACCTGCAAACTGGGAAAGTGCTGGGTTCAAACCAACTGAAAAATTC
_T__D__K__V__P__P__A__N__W__E__S__A__G__F__K__P__T__E__K__F_
OL771200|Pichincha|2
ACTGACAAAGTGCCCCCCGCAAACTGGGAGAGTGCTGGGTACAAACCAACTGAAAAATTC
_T__D__K__V__P__P__A__N__W__E__S__A__G__Y__K__P__T__E__K__F_

| | | | | |

MF978248|Pichincha|2
GCCGCCTTTGACTTCTTTGACGGCGTTGAGAACCCAGCAGCCATGCAACCACCGGGAGGG 480
_A__A__F__D__F__F__D__G__V__E__N__P__A__A__M__Q__P__P__G__G_
MT240490|Imbabura|20
GCCGCCTTTGACTTCTTTGACGGCGTTGAAAACCCAGCGGCCATGCAACCACCGGGAGGG
_A__A__F__D__F__F__D__G__V__E__N__P__A__A__M__Q__P__P__G__G_
MT240491|Imbabura|20
GCCGCCTTTGACTTCTTTGACGGCGTTGAAAACCCAGCAGCCATGCAACCACCGGGAGGG
_A__A__F__D__F__F__D__G__V__E__N__P__A__A__M__Q__P__P__G__G_
MT240492|Imbabura|20
GCCGCCTTTGACTTCTTTGACGGCGTTGAAAACCCAGCAGCCATGCAACCACCGGGAGGG
_A__A__F__D__F__F__D__G__V__E__N__P__A__A__M__Q__P__P__G__G_
MT240493|Imbabura|20
GCCGCCTTTGACTTCTTTGACGGCGTCGAAAACCCAGCAGCCATGCAACCACCGGGAGGG
_A__A__F__D__F__F__D__G__V__E__N__P__A__A__M__Q__P__P__G__G_
MT240494|Pichincha|2
GCCGCCTTTGACTTCTTTGACGGCGTTGAGAACCCAGCAGCTATGCAACCACCGGGAGGG
_A__A__F__D__F__F__D__G__V__E__N__P__A__A__M__Q__P__P__G__G_

MT240495|Pichincha|2

GCCGCCTTCGATTTTTTTGACGGCGGTGAAAACCCAGCAGCCATGCAACCACCGGGAGGG

_A__A__F__D__F__F__D__G__G__E__N__P__A__A__M__Q__P__P__G__G_

MT240496|Tungurahua|

GCCGCCTTTGACTTCTTTGACGGCGTTGAAAACCCAGCTGCTATGCAACCACCGGGAGGG

_A__A__F__D__F__F__D__G__V__E__N__P__A__A__M__Q__P__P__G__G_

MT240497|Tungurahua|

GCCGCCTTTGACTTCTTTGACGGTGTCGAGAACCCAGCAGCCATGCAACCACCGGGAGGG

_A__A__F__D__F__F__D__G__V__E__N__P__A__A__M__Q__P__P__G__G_

MT240498|Tungurahua|

GCCGCCTTTGACTTCTTTGACGGCGTTGAGAACCCAGCAGCCATGCAACCACCGGGAGGG

_A__A__F__D__F__F__D__G__V__E__N__P__A__A__M__Q__P__P__G__G_

MT240499|Tungurahua|

GCCGCCTTTGACTTCTTTGACGGCGTTGAGAACCCAGCAGCCATGCAACCACCGGGAGGG

_A__A__F__D__F__F__D__G__V__E__N__P__A__A__M__Q__P__P__G__G_

MT240500|Tungurahua|

GCCGCCTTTGACTTCTTTGACGGCGTTGAAAACCCAGCAGCCATGCAACCACCGGGAGGG

_A__A__F__D__F__F__D__G__V__E__N__P__A__A__M__Q__P__P__G__G_

MT240501|Tungurahua|

GCCGCCTTTGACTTCTTTGACGGCGTTGAGAACCCAGCAGCCATGCAACCACCGGGAGGG

_A__A__F__D__F__F__D__G__V__E__N__P__A__A__M__Q__P__P__G__G_

MT240502|Tungurahua|

GCCGCCTTTGACTTCTTTGACGGCGTTGAAAACCCAGCAGCCATGCAACCACCGGGAGGA

_A__A__F__D__F__F__D__G__V__E__N__P__A__A__M__Q__P__P__G__G_

MT240503|Tungurahua|

GCCGCCTTTGACTTCTTTGACGGCGTTGAGAACCCAGCAGCCATGCAACCACCGGGAGGG

_A__A__F__D__F__F__D__G__V__E__N__P__A__A__M__Q__P__P__G__G_

MT240504|Tungurahua|

GCCGCCTTTGACTTCTTTGACGGCGTTGAAAACCCAGCAGCCATGCAACCACCGGGAGGA

_A__A__F__D__F__F__D__G__V__E__N__P__A__A__M__Q__P__P__G__G_

MT240505|Azuay|2017|

GCCGCCTTTGACTTCTTTGACGGCGTTGAGAACCCAGCAGCCATGCAACCACCGGGAGGG

_A__A__F__D__F__F__D__G__V__E__N__P__A__A__M__Q__P__P__G__G_

MT240506|Azuay|2017|

GCCGCCTTTGACTTCTTTGACGGCGTTGAGAACCCAGCAGCCATGCAACCACCGGGAGGG

_A__A__F__D__F__F__D__G__V__E__N__P__A__A__M__Q__P__P__G__G_

MT240507|Loja|2017|B

GCCGCCTTTGACTTCTTTGACGGCGTTGAGAACCCAGCAGCCATGCAACCACCGGGAGGG

_A__A__F__D__F__F__D__G__V__E__N__P__A__A__M__Q__P__P__G__G_

MT240508|Loja|2017|B

GCCGCCTTTGACTTCTTTGACGGCGTTGAGAACCCAGCAGCCATGCAACCACCGGGAGGG

_A__A__F__D__F__F__D__G__V__E__N__P__A__A__M__Q__P__P__G__G_

MT240509|Loja|2017|B

GCCGCCTTTGACTTCTTTGACGGCGTTGAGAACCCAGCAGCCATGCAACCACCGGGAGGG

_A__A__F__D__F__F__D__G__V__E__N__P__A__A__M__Q__P__P__G__G_

MT240510|Loja|2017|B

GCCGCCTTTGACTTCTTTGACGGCGTTGAGAACCCAGCAGCCATGCAACCACCGGGAGGG

_A__A__F__D__F__F__D__G__V__E__N__P__A__A__M__Q__P__P__G__G_

MT240511|Loja|2017|B

GCCGCCTTTGACTTCTTTGACGGCGTTGAGAACCCAGCAGCCATGCAACCACCGGGAGGG

_A__A__F__D__F__F__D__G__V__E__N__P__A__A__M__Q__P__P__G__G_

MT240512|Azuay|2016|

GCCGCCTTTGACTTCTTTGACGGCGTTGAAAACCCAGCAGCCATGCAACCACCGGGAGGG

_A__A__F__D__F__F__D__G__V__E__N__P__A__A__M__Q__P__P__G__G_

MT240513|Azuay|2016|

GCCGCCTTTGATTTCTTCGACGGCGTTGAAAACCCAGCCGCCATGCAACCACCGGGAGGG

_A__A__F__D__F__F__D__G__V__E__N__P__A__A__M__Q__P__P__G__G_

MT240514|Azuay|2016|

GCCGCCTTTGATTTCTTCGACGGCGTTGAAAACCCAGCCGCCATGCAACCACCGGGAGGG

_A__A__F__D__F__F__D__G__V__E__N__P__A__A__M__Q__P__P__G__G_

MZ490607|Tungurahua|

GCCGCCTTTGACTTCTTTGACGGCGTTGAAAACCCAGCAGCTATGCAACCACCGGGAGGA

_A__A__F__D__F__F__D__G__V__E__N__P__A__A__M__Q__P__P__G__G_

MZ490608|Tungurahua|

GCCGCCTTTGACTTCTTTGACGGCGTTGAAAACCCAGCGGCTATGCAACCACCGGGAGGA

_A__A__F__D__F__F__D__G__V__E__N__P__A__A__M__Q__P__P__G__G_

MZ490609|Tungurahua|

GCCGCCTTTGACTTCTTTGACGGCGTTGAAAACCCAGCAGCCATGCAACCACCGGGAGGG

_A__A__F__D__F__F__D__G__V__E__N__P__A__A__M__Q__P__P__G__G_

MZ490610|Tungurahua|

GCCGCCTTTGACTTCTTTGACGGTGTTGAGAACCCAGCAGCCATGCGACCACCGGGAGGG

_A__A__F__D__F__F__D__G__V__E__N__P__A__A__M__R__P__P__G__G_

MZ490611|Tungurahua|

GCCGCCTTTGACTTCTTTGACGGTGTTGAGAACCCAGCAGCCATGCGACCACCGGGAGGG

_A__A__F__D__F__F__D__G__V__E__N__P__A__A__M__R__P__P__G__G_

MZ490612|Tungurahua|
GCCGCCTTCGACTTCTTTGACGGCGTTGAAAACCCAGCAGCCATGCAACCACCGGGAGGG
_A__A__F__D__F__F__D__G__V__E__N__P__A__A__M__Q__P__P__G__G_
OL771197|Pichincha|2
GCCGCCTTTGACTTCTTTGACGGCGTTGAGAACCCAGCAGCAATGCAACCACCGGGAGGG
_A__A__F__D__F__F__D__G__V__E__N__P__A__A__M__Q__P__P__G__G_
OL771198|Pichincha|2
GCCGCCTTTGACTTCTTTGACGGCGTTGAGAACCCAGCAGCCATGCAACCACCGGGAGGG
_A__A__F__D__F__F__D__G__V__E__N__P__A__A__M__Q__P__P__G__G_
OL771199|Pichincha|2
GCCGCCTTTGACTTCTTTGACGGCGTTGAAAATCCAGCAGCCATGCAACCACCGGGAGGG
_A__A__F__D__F__F__D__G__V__E__N__P__A__A__M__Q__P__P__G__G_
OL771200|Pichincha|2
GCCGCCTTCGACTTCTTTGACGGCGTTGAAAACCCAGCAGCCATGCAACCACCGGGAGGA
_A__A__F__D__F__F__D__G__V__E__N__P__A__A__M__Q__P__P__G__G_

| | | | | |
MF978248|Pichincha|2
CTCATTAGGTCTCCAAATCAAGCGGAGAGGATAGCCAACCAAACTAACAAACAGGTGAAC 540
_L__I__R__S__P__N__Q__A__E__R__I__A__N__Q__T__N__K__Q__V__N_
MT240490|Imbabura|20
CTCATTAGGTCTCCAAATCAAGCGGAGAGGATAGCCAACCAAACTAACAAACAGGTGAAC
_L__I__R__S__P__N__Q__A__E__R__I__A__N__Q__T__N__K__Q__V__N_
MT240491|Imbabura|20
CTTATTAGGTCTCCAAACCAAGCGGAGAGGATAGCCAACCAAACTAACAAACAGGTGAAC
_L__I__R__S__P__N__Q__A__E__R__I__A__N__Q__T__N__K__Q__V__N_
MT240492|Imbabura|20
CTTATTAGGTCTCCAAACCAAGCGGAGAGGATAGCCAACCAAACTAACAAACAGGTGAAC
_L__I__R__S__P__N__Q__A__E__R__I__A__N__Q__T__N__K__Q__V__N_
MT240493|Imbabura|20
CTCATTAGGTCTCCAAACCAAGCGGAGAGGATAGCCAACCAAACCAACAAACAGGTGAAC
_L__I__R__S__P__N__Q__A__E__R__I__A__N__Q__T__N__K__Q__V__N_
MT240494|Pichincha|2
CTCATTAGATCTCCAAATCAAGCGGAGAGGATAGCCAACCAAACTAACAAACAGGTGAAC
_L__I__R__S__P__N__Q__A__E__R__I__A__N__Q__T__N__K__Q__V__N_
MT240495|Pichincha|2
CTCATTCGGTCTCCAAACCAAGCTGAGAGGATAGCCAACCAAACCAATAAACAGGTGAAT
_L__I__R__S__P__N__Q__A__E__R__I__A__N__Q__T__N__K__Q__V__N_

MT240496|Tungurahua|

CTCATTAGATCCCCAAATCAAGCGGAGAGGATAGCCAACCAAACTAACAAACAGGTGAAC

_L__I__R__S__P__N__Q__A__E__R__I__A__N__Q__T__N__K__Q__V__N_

MT240497|Tungurahua|

CTCATTAGGTCTCCAAACCAAGCGGAGAGGATAGCCAACCAAACTAACAAACAGGTGAGT

_L__I__R__S__P__N__Q__A__E__R__I__A__N__Q__T__N__K__Q__V__S_

MT240498|Tungurahua|

CTCATTAGGTCCCCAAATCAAGCGGAGAGGATAGCCAACCAAACTAACAAACAGGTGAAC

_L__I__R__S__P__N__Q__A__E__R__I__A__N__Q__T__N__K__Q__V__N_

MT240499|Tungurahua|

CTCATTAGGTCTCCAAATCAAGCGGAGAGGATAGCCAACCAAACTAACAAACAGGTGAAC

_L__I__R__S__P__N__Q__A__E__R__I__A__N__Q__T__N__K__Q__V__N_

MT240500|Tungurahua|

CTCACTAGGTCTCCAAATCAAGCGGAGAGGATAGCCAACCAAACTAACAAACAGGTGAAC

_L__T__R__S__P__N__Q__A__E__R__I__A__N__Q__T__N__K__Q__V__N_

MT240501|Tungurahua|

CTCATTAGGTCTCCAAATCAAGCGGAGAGGATAGCCAACCAAACTAACAAACAGGTGAAC

_L__I__R__S__P__N__Q__A__E__R__I__A__N__Q__T__N__K__Q__V__N_

MT240502|Tungurahua|

CTCATCCGGTCCCCAAATCAAGCGGAGAGAATAGCCAACCAGACTAACAAACAAGTGAAC

_L__I__R__S__P__N__Q__A__E__R__I__A__N__Q__T__N__K__Q__V__N_

MT240503|Tungurahua|

CTCATTAGGTCTCCAAATCAAGCGGAGAGGATAGCCAACCAAACTAACAAACAGGTGAAC

_L__I__R__S__P__N__Q__A__E__R__I__A__N__Q__T__N__K__Q__V__N_

MT240504|Tungurahua|

CTCATCCGGTCCCCAAATCAAGCGGAGAGAATAGCCAACCAAACTAACAAACAAGTGAAC

_L__I__R__S__P__N__Q__A__E__R__I__A__N__Q__T__N__K__Q__V__N_

MT240505|Azuay|2017|

CTCATTAGGTCTCCAAACCAAGCGGAGAGGATAGCCAACCAAACTAACAAACAGGTGAAC

_L__I__R__S__P__N__Q__A__E__R__I__A__N__Q__T__N__K__Q__V__N_

MT240506|Azuay|2017|

CTCATTAGGTCTCCAAACCAAGCGGAGAGGATAGCCAACCAAACTAACAAACAGGTGAAC

_L__I__R__S__P__N__Q__A__E__R__I__A__N__Q__T__N__K__Q__V__N_

MT240507|Loja|2017|B

CTCATTAGGTCTCCAAACCAAGCGGAGAGGATAGCCAACCAAACTAACAAACAGGTGAAC

_L__I__R__S__P__N__Q__A__E__R__I__A__N__Q__T__N__K__Q__V__N_

MT240508|Loja|2017|B

CTCATTAGGTCTCCAAACCAAGCGGAGAGGATAGCCAACCAAACTAACAAACAGGTGAAC

_L__I__R__S__P__N__Q__A__E__R__I__A__N__Q__T__N__K__Q__V__N_

MT240509|Loja|2017|B

CTCATTAGGTCTCCAAACCAAGCGGAGAGGATAGCCAACCAAACTAACAAACAGGTGAAC

_L__I__R__S__P__N__Q__A__E__R__I__A__N__Q__T__N__K__Q__V__N_

MT240510|Loja|2017|B

CTCATAAGGTCTCCAAACCAAGCGGAGAGGATAGCCAACCAAACTAACAAACAGGTGAAC

_L__I__R__S__P__N__Q__A__E__R__I__A__N__Q__T__N__K__Q__V__N_

MT240511|Loja|2017|B

CTCATTAGGTCTCCAAACCAAGCGGAGAGGATAGCCAACCAAACTAACAAACAGGTGAAC

_L__I__R__S__P__N__Q__A__E__R__I__A__N__Q__T__N__K__Q__V__N_

MT240512|Azuay|2016|

CTCGTTCGGTCTCCAAACCAAGCGGAGAGGATAGCCAACCAAACGAACAAACAGGTGAAC

_L__V__R__S__P__N__Q__A__E__R__I__A__N__Q__T__N__K__Q__V__N_

MT240513|Azuay|2016|

CTCATTCGGTCTCCAAACCAAGCGGAGAGAATAGCCAACCAAACCAACAAACAGGTGAAC

_L__I__R__S__P__N__Q__A__E__R__I__A__N__Q__T__N__K__Q__V__N_

MT240514|Azuay|2016|

CTCATTCGGTCTCCAAACCAAGCGGAGAGAATAGCCAACCAAACCAACAAACAGGTGAAC

_L__I__R__S__P__N__Q__A__E__R__I__A__N__Q__T__N__K__Q__V__N_

MZ490607|Tungurahua|

CTCATTAGATCCCCAAATCAAGCGGAGAGGATAGCCAATCAAACTAACAAACAGGTGAAT

_L__I__R__S__P__N__Q__A__E__R__I__A__N__Q__T__N__K__Q__V__N_

MZ490608|Tungurahua|

CTCATTAGATCCCCAAATCAAGCGGAGAGGATAGCCAACCAAACTAACAAACAGGTGAAC

_L__I__R__S__P__N__Q__A__E__R__I__A__N__Q__T__N__K__Q__V__N_

MZ490609|Tungurahua|

CTCATTAGATCTCCAAACCAAGCGGAGAGGATAGCCAACCAAACTAACAAACAGGTGAAC

_L__I__R__S__P__N__Q__A__E__R__I__A__N__Q__T__N__K__Q__V__N_

MZ490610|Tungurahua|

CTCATTAGGTCTCCTAATCAGGCGGAGAGGATAGCCAACCAAACTAACAAACAGGTGAAC

_L__I__R__S__P__N__Q__A__E__R__I__A__N__Q__T__N__K__Q__V__N_

MZ490611|Tungurahua|

CTCATTAGGTCTCCTAATCAGGCGGAGAGGATAGCCAACCAAACTAACAAACAGGTGAAC

_L__I__R__S__P__N__Q__A__E__R__I__A__N__Q__T__N__K__Q__V__N_

MZ490612|Tungurahua|

CTCATTCGGTCTCCAAACCAAGCTGAGAGGATCGCTAACCAAACTAACAAACAGGTGAAC

_L__I__R__S__P__N__Q__A__E__R__I__A__N__Q__T__N__K__Q__V__N_

OL771197|Pichincha|2

CTCATTAGATTTCCAAACCAAGCGGAGAGGATAGCCAACCAAACTAACAAACAGGTGAAC

_L__I__R__F__P__N__Q__A__E__R__I__A__N__Q__T__N__K__Q__V__N_

OL771198|Pichincha|2

CTCATTAGATTTCCAAACCAAGCGGAGAGGATAGCCAACCAAACTAACAAACAGGTGAAC

_L__I__R__F__P__N__Q__A__E__R__I__A__N__Q__T__N__K__Q__V__N_

OL771199|Pichincha|2

CTCATCAGGTCTCCAAACCAAGCGGAGAGGATAGCCAACCAAACTAACAAACAGGTGAAC

_L__I__R__S__P__N__Q__A__E__R__I__A__N__Q__T__N__K__Q__V__N_

OL771200|Pichincha|2

CTCATTCGGTCTCCAAACCAAGCTGAGAGGATAGCCAACCAAACTAACAAACAGGTGAAC

_L__I__R__S__P__N__Q__A__E__R__I__A__N__Q__T__N__K__Q__V__N_

| | | | | |

MF978248|Pichincha|2

CTCTTCCAAACCGCAGCCCAGGGAAATAACTTGGCCTCCAACTCTGCATTCATAACCAAG 600

_L__F__Q__T__A__A__Q__G__N__N__L__A__S__N__S__A__F__I__T__K_

MT240490|Imbabura|20

CTCTTCCAAACTGCAGCCCAGGGGAATAACTTGGCCTCCAACTCAGCGTTCATAACCAAG

_L__F__Q__T__A__A__Q__G__N__N__L__A__S__N__S__A__F__I__T__K_

MT240491|Imbabura|20

CTCTTCCAAACTGCAGCCCAGGGAAATAACTTGGCCTCCAACTCAGCGTTTATAACCAAG

_L__F__Q__T__A__A__Q__G__N__N__L__A__S__N__S__A__F__I__T__K_

MT240492|Imbabura|20

CTCTTCCAAACTGCAGCCCAGGGAAATAACCTGGCCTCCAACTCAGCGTTTATAACCAAG

_L__F__Q__T__A__A__Q__G__N__N__L__A__S__N__S__A__F__I__T__K_

MT240493|Imbabura|20

CTCTTCCAAACTGCAGCCCAGGGGAATAACTTGGCCTCCAACTCAGCGTTTATAACCAAG

_L__F__Q__T__A__A__Q__G__N__N__L__A__S__N__S__A__F__I__T__K_

MT240494|Pichincha|2

CTCTTCCAAACCGCAGCCCAGGGGAATAACTTGGCCTCGAACTCTGCATTCATAACCAAG

_L__F__Q__T__A__A__Q__G__N__N__L__A__S__N__S__A__F__I__T__K_

MT240495|Pichincha|2

CTCTTCCAAACTGCAGCTCAGGGGAACAACTTGGCTTCCAACTCTGCATTCATAACCAAG

_L__F__Q__T__A__A__Q__G__N__N__L__A__S__N__S__A__F__I__T__K_

MT240496|Tungurahua|

CTCTTCCAAACCGCAGCCCAGGGGAATAACTTGGCCTCTAACTCCGCATTCATAACCAAG

_L__F__Q__T__A__A__Q__G__N__N__L__A__S__N__S__A__F__I__T__K_

MT240497|Tungurahua|

CTCTTCCAAACTGCAGCCCAGGGGAATAACTTGGCCTCCAACTCCGCATTCATAACCAAG

_L__F__Q__T__A__A__Q__G__N__N__L__A__S__N__S__A__F__I__T__K_

MT240498|Tungurahua|

CTCTTCCAAACCGCAGCCCAGGGAAATAACTTGGCCTCCAATTCCGCATTCATAACCAAG

_L__F__Q__T__A__A__Q__G__N__N__L__A__S__N__S__A__F__I__T__K_

MT240499|Tungurahua|

CTCTTCCAAACCGCAGCCCAGGGAAATAACTTGGCCTCCAATTCCGCATTCATAACCAAG

_L__F__Q__T__A__A__Q__G__N__N__L__A__S__N__S__A__F__I__T__K_

MT240500|Tungurahua|

CTCTTCCAAACCGCAGCTCAGGGAACTAACTTGGCCTCCAACTCCGCATTCATAACCAAG

_L__F__Q__T__A__A__Q__G__T__N__L__A__S__N__S__A__F__I__T__K_

MT240501|Tungurahua|

CTCTTCCAAACCGCAGCCCAGGGAAATAACTTGGCCTCCAACTCCGCATTCATAACCAAG

_L__F__Q__T__A__A__Q__G__N__N__L__A__S__N__S__A__F__I__T__K_

MT240502|Tungurahua|

CTCTTCCAAACTGCAGCTCAGGGGAACAACTTGGCCTCCAACTCTGCATTCATAACCAAG

_L__F__Q__T__A__A__Q__G__N__N__L__A__S__N__S__A__F__I__T__K_

MT240503|Tungurahua|

CTCTTCCAAACCGCAGCCCAGGGAAATAACTTGGCCTCCAACTCCGCATTCATAACCAAG

_L__F__Q__T__A__A__Q__G__N__N__L__A__S__N__S__A__F__I__T__K_

MT240504|Tungurahua|

CTCTTCCAAACTGCAGCTCAGGGGAACAACTTGGCCTCCAACTCTGCATTCATAACCAAG

_L__F__Q__T__A__A__Q__G__N__N__L__A__S__N__S__A__F__I__T__K_

MT240505|Azuay|2017|

CTCTTCCAAGCTGCTGCCCAGGGGAATAACTTGGCTTCCAACTCAGCATTCATAACCAAG

_L__F__Q__A__A__A__Q__G__N__N__L__A__S__N__S__A__F__I__T__K_

MT240506|Azuay|2017|

CTCTTCCAAGCTGCTGCCCAGGGGAATAACTTGGCTTCCAACTCAGCATTCATAACCAAG

_L__F__Q__A__A__A__Q__G__N__N__L__A__S__N__S__A__F__I__T__K_

MT240507|Loja|2017|B

CTCTTCCAAGCTGCTGCCCAGGGGAATAACTTGGCTTCCAACTCAGCATTCATAACCAAG

_L__F__Q__A__A__A__Q__G__N__N__L__A__S__N__S__A__F__I__T__K_

MT240508|Loja|2017|B

CTCTTCCAAGCTGCTGCCCAGGGGAATAACTTGGCTTCCAACTCAGCATTCATAACCAAG

_L__F__Q__A__A__A__Q__G__N__N__L__A__S__N__S__A__F__I__T__K_

MT240509|Loja|2017|B

CTCTTCCAAGCTGCTGCCCAGGGGAATAACTTGGCTTCCAACTCAGCATTCATAACCAAG

_L__F__Q__A__A__A__Q__G__N__N__L__A__S__N__S__A__F__I__T__K_

MT240510|Loja|2017|B

CTCTTCCAAGCTGCTGCCCAGGGGAATAACTTGGCTTCCAACTCAGCATTCATAACCAAG

_L__F__Q__A__A__A__Q__G__N__N__L__A__S__N__S__A__F__I__T__K_

MT240511|Loja|2017|B

CTCTTCCAAGCTGCTGCCCAGGGGAATAACTTGGCTTCCAACTCAGCATTCATAACCAAG

_L__F__Q__A__A__A__Q__G__N__N__L__A__S__N__S__A__F__I__T__K_

MT240512|Azuay|2016|

CTCTTCCAAACTGCAGCTCAGGGAAATAACTTGGCATCCAACTCTGCATTCATAACCAAG

_L__F__Q__T__A__A__Q__G__N__N__L__A__S__N__S__A__F__I__T__K_

MT240513|Azuay|2016|

CTCTTCCAAACTGCAGCCCAGGGGAATAACTTGGCCTCCAATTCTGCATTCATAACCAAG

_L__F__Q__T__A__A__Q__G__N__N__L__A__S__N__S__A__F__I__T__K_

MT240514|Azuay|2016|

CTCTTCCAAACTGCAGCCCAGGGGAATAACTTGGCCTCCAATTCTGCATTCATAACCAAG

_L__F__Q__T__A__A__Q__G__N__N__L__A__S__N__S__A__F__I__T__K_

MZ490607|Tungurahua|

CTCTTCCAAACCGCAGCCCAGGGGAATAACTTGGCCTCTAACTCCGCATTCATAACCAAG

_L__F__Q__T__A__A__Q__G__N__N__L__A__S__N__S__A__F__I__T__K_

MZ490608|Tungurahua|

CTCTTCCAAACCGCAGCCCAGGGGAATAACTTGGCCTCTAACTCCGCATTCATAACCAAG

_L__F__Q__T__A__A__Q__G__N__N__L__A__S__N__S__A__F__I__T__K_

MZ490609|Tungurahua|

CTCTTCCAAACCGCAGCTCAAGGGAATAACTTGGCCTCCAACTCCGCATTCATAACCAAG

_L__F__Q__T__A__A__Q__G__N__N__L__A__S__N__S__A__F__I__T__K_

MZ490610|Tungurahua|

CTCTTCCAAGCCGCAGCCCAGGGGAACAACTTGGCTTCTAACTCCGCATTCATAACTAAG

_L__F__Q__A__A__A__Q__G__N__N__L__A__S__N__S__A__F__I__T__K_

MZ490611|Tungurahua|

CTCTTCCAAACCGCAGCCCAGGGGAACAACTTGGCTTCTAACTCCGCATTCATAACCAAG

_L__F__Q__T__A__A__Q__G__N__N__L__A__S__N__S__A__F__I__T__K_

MZ490612|Tungurahua|

CTCTTCCAAACTGCAGCTCAGGGGAACAACTTGGCCTCCAACTCCGCATTCATAACCAAG

_L__F__Q__T__A__A__Q__G__N__N__L__A__S__N__S__A__F__I__T__K_

OL771197|Pichincha|2

CTCTTCCAAGCTGCTGCCCAGGGAAATAACTTGGCTTCCAACTCAGCATTCATAACCAAG

_L__F__Q__A__A__A__Q__G__N__N__L__A__S__N__S__A__F__I__T__K_

OL771198|Pichincha|2
CTCTTCCAAGCTGCTGCCCAGGGAAATAACTTGGCTTCCAACTCAGCATTCATAACCAAG
_L__F__Q__A__A__A__Q__G__N__N__L__A__S__N__S__A__F__I__T__K_
OL771199|Pichincha|2
CTCTTCCAAACCGCGGCCCAGGGGAACAACTTGGCCTCCAACTCCGCATTCATAACCAAG
_L__F__Q__T__A__A__Q__G__N__N__L__A__S__N__S__A__F__I__T__K_
OL771200|Pichincha|2
CTCTTCCAAACTGCAGCTCAGGGGAACAACTTGGCCTCCAACTCCGCATTTATAACCAAG
_L__F__Q__T__A__A__Q__G__N__N__L__A__S__N__S__A__F__I__T__K_

| | | | |
MF978248|Pichincha|2 GGGCAGATTTCAACGTCCACTCCATCTATCCAGTTTCTCCCATCCCCAGAGTAG
654
_G__Q__I__S__T__S__T__P__S__I__Q__F__L__P__S__P__E__*_
MT240490|Imbabura|20
GGGCAGATCTCAACGTCCACTCCATCCATCCAGTTTCTTCCATCCCCCGAGTAG
_G__Q__I__S__T__S__T__P__S__I__Q__F__L__P__S__P__E__*_
MT240491|Imbabura|20
GGGCAGATCTCAACGTCCACACCATCCATCCAGTTTCTTCCATCCCCCGAGTAG
_G__Q__I__S__T__S__T__P__S__I__Q__F__L__P__S__P__E__*_
MT240492|Imbabura|20
GGGCAGATCTCAACGTCCACACCATCCATCCAGTTTCTTCCATCCCCCGAGTAG
_G__Q__I__S__T__S__T__P__S__I__Q__F__L__P__S__P__E__*_
MT240493|Imbabura|20
GGGCAGGTCTCAACGTCCACTCCATCCATCCAGTTTCTTCCATCCCCCGATTAG
_G__Q__V__S__T__S__T__P__S__I__Q__F__L__P__S__P__D__*_
MT240494|Pichincha|2 GGGCAAATTTCAACGTCCACTCCATCCATCCAGTTTCTTCCATCCCCCGAGTAG
_G__Q__I__S__T__S__T__P__S__I__Q__F__L__P__S__P__E__*_
MT240495|Pichincha|2 GGGCAGATCTCAACTTCCACTCCATCCATCCAGTTTCTTCCATCCCCCGAGTAG
_G__Q__I__S__T__S__T__P__S__I__Q__F__L__P__S__P__E__*_
MT240496|Tungurahua|
GGGCAGATCTCAACGTCCACTCCATCCATCCAGTTTCTTCCATCTCCCGAGTAG
_G__Q__I__S__T__S__T__P__S__I__Q__F__L__P__S__P__E__*_
MT240497|Tungurahua|
GGGCAGATCTCAACGTCCACTCCATCCATCCAGTTTCTTCCATCCCCCGAGTAG
_G__Q__I__S__T__S__T__P__S__I__Q__F__L__P__S__P__E__*_
MT240498|Tungurahua|
GGGCAGATCTCAACGTCCACTCCATCCATCCAGTTTCTTCCATCCCCCGAGTAG

_G__Q__I__S__T__S__T__P__S__I__Q__F__L__P__S__P__E__*_

MT240499|Tungurahua|

GGGCAGATCTCAACGTCCACTCCATCCATCCAGTTTCTTCCATCCCCCGAGTAG

_G__Q__I__S__T__S__T__P__S__I__Q__F__L__P__S__P__E__*_

MT240500|Tungurahua|

GGGCAGATCTCAACGTCCACTCCATCCATTCAGTTTCTTCCATCCCCCGAGTAG

_G__Q__I__S__T__S__T__P__S__I__Q__F__L__P__S__P__E__*_

MT240501|Tungurahua|

GGGCAGATCTCAACGTCCACTCCATCCATCCAGTTTCTTCCATCCCCCGAGTAG

_G__Q__I__S__T__S__T__P__S__I__Q__F__L__P__S__P__E__*_

MT240502|Tungurahua|

GGGCAGATCTCAACGTCCACTCCATCCATCCAGTTTCTTCCGTCTCCCGAGTAG

_G__Q__I__S__T__S__T__P__S__I__Q__F__L__P__S__P__E__*_

MT240503|Tungurahua|

GGGCAGATCTCAACGTCCACTCCATCCATCCAGTTTCTTCCATCCCCCGAGTAG

_G__Q__I__S__T__S__T__P__S__I__Q__F__L__P__S__P__E__*_

MT240504|Tungurahua|

GGGCAGATCTCAACGTCCACTCCATCCATCCAGTTTCTTCCGTCTCCCGAGTAG

_G__Q__I__S__T__S__T__P__S__I__Q__F__L__P__S__P__E__*_

MT240505|Azuay|2017|

GGGCAGATCTCAACGTCCACTCCATCCATCCAGTTTCTTCCATCCCCCGAGTAG

_G__Q__I__S__T__S__T__P__S__I__Q__F__L__P__S__P__E__*_

MT240506|Azuay|2017|

GGGCAGATCTCAACGTCCACTCCATCCATCCAGTTTCTTCCATCCCCCGAGTAG

_G__Q__I__S__T__S__T__P__S__I__Q__F__L__P__S__P__E__*_

MT240507|Loja|2017|B

GGGCAGATCTCAACGTCCACTCCATCCATCCAGTTTCTTCCATCCCCCGAGTAG

_G__Q__I__S__T__S__T__P__S__I__Q__F__L__P__S__P__E__*_

MT240508|Loja|2017|B

GGGCAGATCTCAGCGTCCACTCCATCCATCCAGTTTCTTCCATCCCCCGAGTAG

_G__Q__I__S__A__S__T__P__S__I__Q__F__L__P__S__P__E__*_

MT240509|Loja|2017|B

GGGCAGATCTCAACGTCCACTCCATCCATCCAGTTTCTTCCATCCCCCGAGTAG

_G__Q__I__S__T__S__T__P__S__I__Q__F__L__P__S__P__E__*_

MT240510|Loja|2017|B

GGGCAGATCTCAGCGTCCACTCCATCCATCCAGTTTCTTCCATCCCCCGAGTAG

_G__Q__I__S__A__S__T__P__S__I__Q__F__L__P__S__P__E__*_

MT240511|Loja|2017|B

GGGCAGATCTCAACGTCCACTCCATCCATCCAGTTTCTTCCATCCCCCGAGTAG

_G__Q__I__S__T__S__T__P__S__I__Q__F__L__P__S__P__E__*_

MT240512|Azuay|2016|

GGGCAGATCTCAACGTCCACTCCATCCATTCAGTTTCTTCCATCTCCAGAATAG

_G__Q__I__S__T__S__T__P__S__I__Q__F__L__P__S__P__E__*_

MT240513|Azuay|2016|

GGGCAGATCTCGACGTCCACTCCATCCATCCAGTTTCTTCCGTCTCCAGAGTAG

_G__Q__I__S__T__S__T__P__S__I__Q__F__L__P__S__P__E__*_

MT240514|Azuay|2016|

GGGCAGATCTCGACGTCCACTCCATCCATCCAGTTTCTTCCGTCTCCAGAGTAG

_G__Q__I__S__T__S__T__P__S__I__Q__F__L__P__S__P__E__*_

MZ490607|Tungurahua| GGGCAGATCTCAACGTCCACTCCATCCATCCAGTTTCTTCCATCCCCA------

_G__Q__I__S__T__S__T__P__S__I__Q__F__L__P__S__P__-__-_

MZ490608|Tungurahua| GGGCAGATCTCAACGTCCACTCCATCCATCCAGTTTCTTCCATC----------

_G__Q__I__S__T__S__T__P__S__I__Q__F__L__P__#__-__-__-_

MZ490609|Tungurahua| GGGCAGATCTCAACGTCCACTCCATCCATCCAGTTTCTTCCATCCC--------

_G__Q__I__S__T__S__T__P__S__I__Q__F__L__P__S__#__-__-_

MZ490610|Tungurahua| GGGCAGATCTCAACGTCCACTCCATCCATCCAGTTTCTTCCATCCCCAG-----

_G__Q__I__S__T__S__T__P__S__I__Q__F__L__P__S__P__#__-_

MZ490611|Tungurahua| GGGCAGATCTCAACGTCCACTCCATCCATCCAGTTTCTTCCATCCC--------

_G__Q__I__S__T__S__T__P__S__I__Q__F__L__P__S__#__-__-_

MZ490612|Tungurahua|

GGGCAGATCTCCACATCCACTCCATCCATCCAGTTTCTTCCATCCCCAGAATAG

_G__Q__I__S__T__S__T__P__S__I__Q__F__L__P__S__P__E__*_

OL771197|Pichincha|2 GGGCAGATCTCAACGTCCACTCCATCCATCCAGTTTCTTCCATCCCCAGAATAG

_G__Q__I__S__T__S__T__P__S__I__Q__F__L__P__S__P__E__*_

OL771198|Pichincha|2 GGGCAGATCTCAACGTCCACTCCATCCATCCAGTTTCTTCCATCCCCAGAATAA

_G__Q__I__S__T__S__T__P__S__I__Q__F__L__P__S__P__E__*_

OL771199|Pichincha|2 GGGCAGATCTCAACGTCCACTCCATCCATCCAGTTTCTTCCATCCCCAGAATAG

_G__Q__I__S__T__S__T__P__S__I__Q__F__L__P__S__P__E__*_

OL771200|Pichincha|2 GGGCAGATCTCAACGTCCACTCCATCCATCCAGTTTCTTCCATCCCCAGAATAG

_G__Q__I__S__T__S__T__P__S__I__Q__F__L__P__S__P__E__*_

Figure S1. Babaco mosaic virus coat proteins alignment with the overall structure.

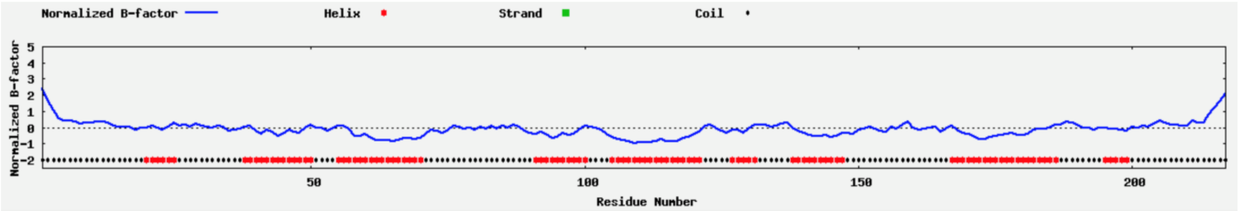

Figure S2. Normalized B-factor for the babaco mosaic virus coat protein model generated with I-TASSER.

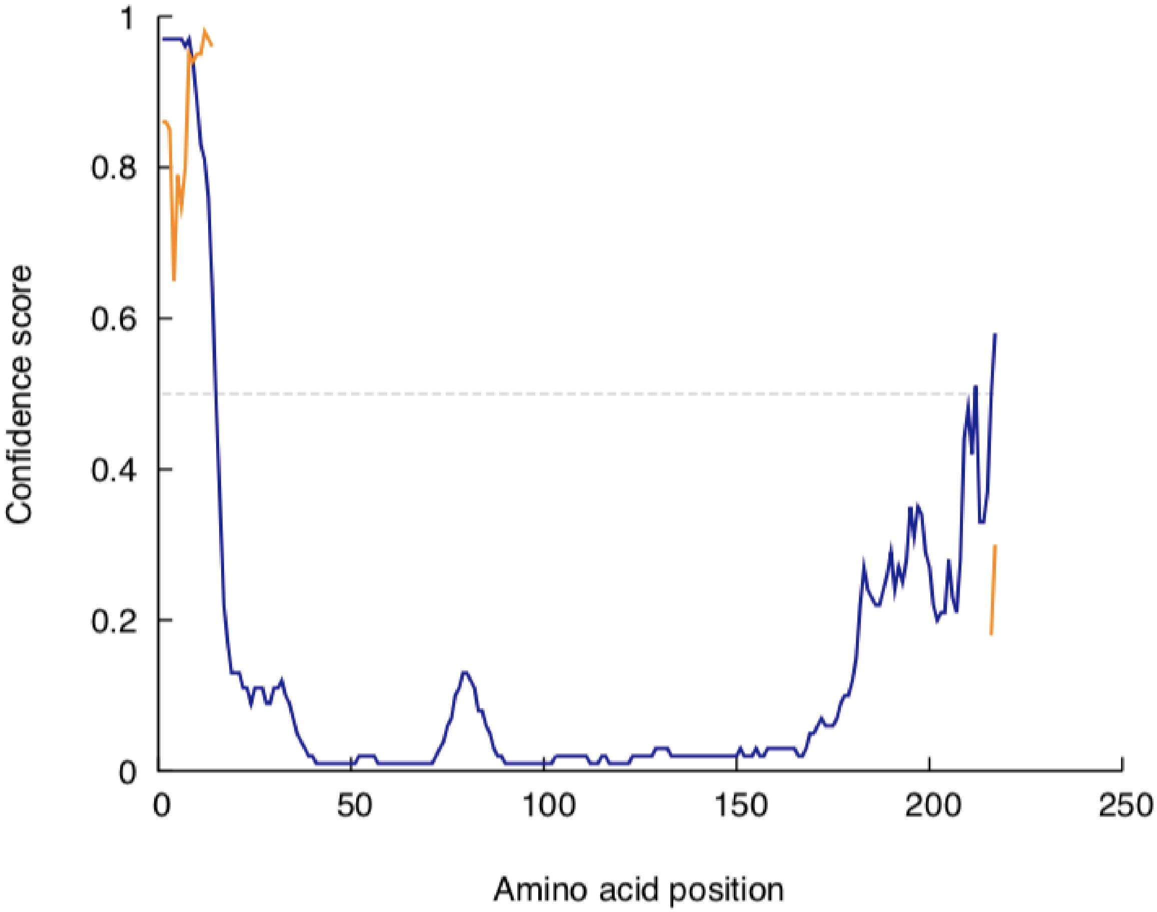

Figure S3. A probability estimate of the disorder per residue with DISOPRED for the coat protein of babaco mosaic virus. Both C and N terminals show prevalence of disorder, in this case an indicator of the flexibility of the BabMV CP.

A

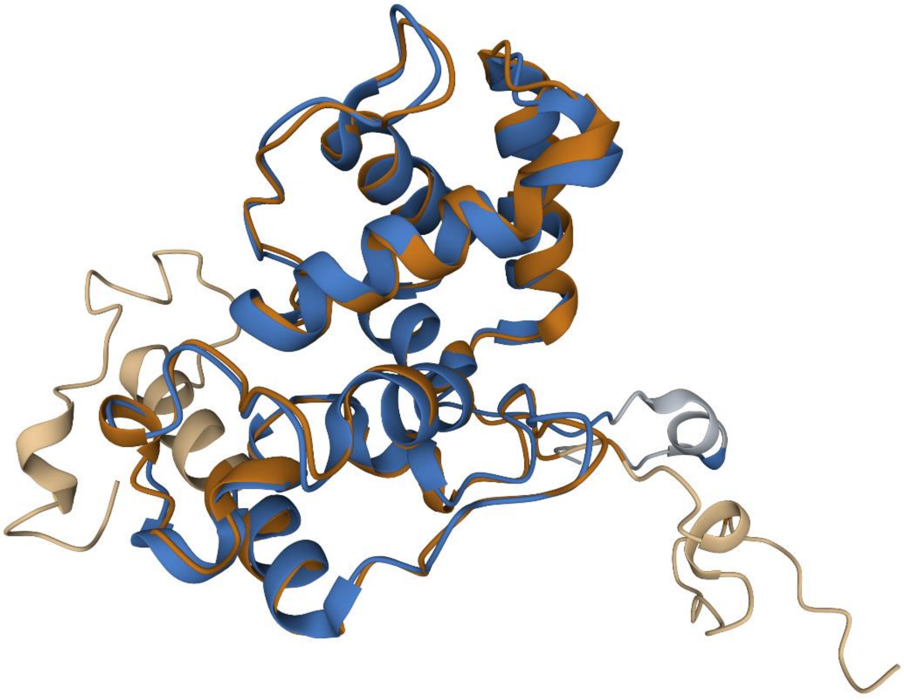

B

```
1     ------MSGKSESSN-------TGNSPFPNLTKETMASFNFKPSSNLLPS    37
11    IAFPAI-------TQEQMSSIK-----------------VD-PTSNLLPS    35
38    EEELKTISTLLVAAKIPSASTTIVAMDLVNFCYDNGSSVYTVISGESSVT    87
36    QEQLKSVSTLMVAAKVPAASVTTVALELVNFCYDNGSSAYTTVTGPSSIP    85
88    GVTLAQIASIVKASGTSLRKFCRFFAPVIWNLRTDKVPPANWESAGYKPT    137
86    EISLAQLASIVKASGTSLRKFCRYFAPIIWNLRTDKMAPANWEASGYKPS    135
138   EKFAAFDFFDGVENPAAMQPPGGLIRSPNQAERIANQTNKQVNLFQTAAQ    187
136   AKFAAFDFFDGVENPAAMQPPSGLTRSPTQEERIANATN-----------    174
188   GNNLASNSAFITKGQISTSTPSIQFLPSPE                        217
      ------------------------------
```

Figure S4. Three-dimensional (**A**) and sequence (**B**) alignment between the coat proteins of babaco mosaic virus (orange) and papaya mosaic virus (blue).

Table S1. Structure quality assessment with ERRAT for the babaco mosaic virus coat protein

Protein	Overall Quality Factor before molecular dynamics	Overall Quality Factor after molecular dynamics (%)		
		Cluster 1	Cluster 2	Cluster 3
CP	76.442	95.939	97.980	97.423

Table S2. Structure quality assessment with Verify 3D for the babaco mosaic virus coat protein

Protein	3D-1D score ≥ 0.2, before molecular dynamics (%)	3D-1D score ≥ 0.2, after molecular dynamics (%)		
		Cluster 1	Cluster 2	Cluster 3
CP	55.700	95.939	97.980	97.423

Table S3. Structure quality assessment with ProCheck for the babaco mosaic virus coat protein. Ramachandran plot residue analysis

	Core (%)	Allowed (%)	Generous (%)	Disallowed (%)
Residues by class before MD	86.1	11.8	0	2.1
Residues by class after MD- Cluster 1	88.8	9.1	1.1	1.1
Residues by class after MD-Cluster 2	88.2	11.2	0	0.5
Residues by class after MD-Cluster 3	88.8	9.1	1.1	1.1

Table S4. Predicted linear epitopes of the coat proteins of BabMV and PapMV

			**BabMV**		
N°	Start	End	Peptide	Number of residues	Score
1	1	26	MSGKSESSNTGNSPFPNLTKETMASF	26	0.869
2	185	217	AAQGNNLASNSAFITKGQISTSTPSIQFLPSPE	33	0.804
3	46	57	TLLVAAKIPSAS	12	0.720
4	81	90	SGESSVTGVT	10	0.660
5*	121	125	TDKVP	5	0.575
6*	128	139	NWESAGYKPTEK	12	0.528
7*	157	168	PPGGLIRSPNQA	12	0.515
			**PapMV**		
N°	Start	End	Peptide	Number of residues	Score
1	11	28	IAFPAITQEQMSSIKVDP	18	0.884
2*	154	174	QPPSGLTRSPTQEERIANATN	21	0.660
3*	120	137	DKMAPANWEASGYKPSAK	18	0.659
4	73	91	SAYTTVTGPSSIPEISLAQ	19	0.630

*: common structural linear epitope region in BabMV and PapMV. Score is defined as a protrusion index value (PI) averaged over epitope residues.

Table S5. Predicted discontinuous epitopes of the coat proteins of BabMV and PapMV

	**BabMV**		
N°	Residues	Number of residues	Score
1	A:M1, A:S2, A:G3, A:K4, A:S5, A:E6, A:S7, A:S8, A:N9, A:T10, A:G11, A:N12, A:S13, A:P14, A:F15, A:P16, A:N17, A:L18, A:T19, A:K20, A:E21, A:T22, A:M23, A:A24, A:S25, A:F26, A:N27	27	0.855
2	A:F182, A:A185, A:A186, A:Q187, A:G188, A:N189, A:N190, A:L191, A:A192, A:S193, A:N194, A:S195, A:A196, A:F197, A:I198, A:T199, A:K200, A:G201, A:Q202, A:I203, A:S204, A:T205, A:S206, A:T207, A:P208, A:S209, A:I210, A:Q211, A:F212, A:L213, A:P214, A:S215, A:P216, A:E217	34	0.797
3	A:K42, A:T43, A:T46, A:L47, A:L48, A:V49, A:A50, A:A51, A:K52, A:I53, A:P54, A:S55, A:A56, A:S57, A:I60, A:S81, A:G82, A:E83, A:S84, A:S85, A:V86, A:T87, A:G88, A:V89, A:T90, A:Q93	26	0.671

4*	A:T121, A:D122, A:K123, A:V124, A:P125, A:W129, A:E130, A:S131, A:A132, A:G133, A:Y134, A:K135, A:P136, A:T137, A:E138, A:K139, A:L161, A:I162, A:R163, A:S164, A:P165, A:N166, A:Q167, A:A168, A:I171	25	0.547

PapMV

N°	Residues	Number of residues	Score
1*	A:D120, A:K121, A:M122, A:A123, A:P124, A:A125, A:N126, A:W127, A:E128, A:A129, A:S130, A:G131, A:Y132, A:K133, A:P134, A:S135, A:A136, A:K137, A:Q154, A:P155, A:P156, A:S157, A:G158, A:L159, A:T160, A:R161, A:S162, A:P163, A:T164, A:Q165, A:E166, A:E167, A:R168, A:I169, A:A170, A:A172, A:T173, A:N174	38	0.667
2	A:I11, A:A12, A:F13, A:P14, A:A15, A:I16, A:T17, A:Q18, A:E19, A:Q20, A:M21, A:S22, A:S23, A:I24, A:K25, A:V26, A:D27, A:P28	18	0.884
3	A:S73, A:A74, A:Y75	3	0.563
4	A:T76, A:T77, A:V78, A:T79, A:G80, A:P81, A:S82, A:S83, A:I84, A:P85, A:E86, A:I87, A:S88, A:A90, A:Q91, A:S94	16	0.652
5	A:T44, A:L45, A:V47, A:A48, A:A49, A:K50, A:V51, A:P52, A:A53, A:A54, A:S55	11	0.738

*: common structural region of discontinuous antibody epitope in BabMV and PapMV. Score is defined as a protrusion index value (PI) averaged over epitope residues.
